# Predicting flows through microfluidic circuits with fluid walls

**DOI:** 10.1038/s41378-021-00322-6

**Published:** 2021-11-18

**Authors:** Cyril Deroy, Nicholas Stovall-Kurtz, Federico Nebuloni, Cristian Soitu, Peter R. Cook, Edmond J. Walsh

**Affiliations:** 1 Department of Engineering Science, Osney Thermo-Fluids Laboratory, University of Oxford, Oxford, OX2 0ES UK; 2 Sir William Dunn School of Pathology, University of Oxford, Oxford, OX1 3RE UK; 3 Cold Spring Harbor Laboratory, Cold Spring Harbor, NY USA

**Keywords:** Engineering, Physics

## Abstract

The aqueous phase in traditional microfluidics is usually confined by solid walls; flows through such systems are often predicted accurately. As solid walls limit access, open systems are being developed in which the aqueous phase is partly bounded by fluid walls (interfaces with air or immiscible liquids). Such fluid walls morph during flow due to pressure gradients, so predicting flow fields remains challenging. We recently developed a version of open microfluidics suitable for live-cell biology in which the aqueous phase is confined by an interface with an immiscible and bioinert fluorocarbon (FC40). Here, we find that common medium additives (fetal bovine serum, serum replacement) induce elastic no-slip boundaries at this interface and develop a semi-analytical model to predict flow fields. We experimentally validate the model’s accuracy for single conduits and fractal vascular trees and demonstrate how flow fields and shear stresses can be controlled to suit individual applications in cell biology.

## Introduction

Flow is important in many biomedical applications, as cell survival and behavior depend critically on it.^1^ It is also essential in organ-on-a-chip devices^2–4^ where cells are perfused continuously to mimic in vivo conditions and when studying the effects of transient shear stress on cells.^5^ Defining flow fields in these systems is thus essential. The aqueous phase in conventional microfluidic devices is typically surrounded by solid walls (made, for example, of polydimethylsiloxane, PDMS), and flows through them can usually be predicted.^6–8^ For example, equations based on pipe flow can be adapted for conduits with arbitrary cross-sections^9^ and to model vascular circuits with branches of varying widths.^10–13^ As solid walls restrict access, open microfluidics are being developed where parts of walls are replaced by interfaces with air or immiscible liquids^14–16^ and equations describing rivulet^17^ multiphase^18^ and droplet-based flows^19^ through conduits with free surfaces have been described.

Recently, an open system termed fluid-walled microfluidics was proved to be particularly suited to biomedical applications. Circuits are built using just a cell culture medium and Petri dishes that biologists use daily, in addition to the bioinert cell-friendly and immiscible fluorocarbon FC40; the two liquids sitting in virgin dishes are reshaped into circuits in seconds.^20–22^ The medium in these circuits is confined by FC40 walls held by interfacial forces, and aqueous conduits have cross-sectional profiles of segments of circles that morph as pressures change during flow. Although predicting flows through such morphing cross-sections is challenging, asymptotic methods have been introduced and validated using numerical simulations for transient (passively pumped) systems.^23^ However, the development of simpler methods and their experimental validation are still necessary. Here, we characterize how fluid walls change during flow through a simple open-ended conduit. Surprisingly, we find that a common medium component—fetal bovine serum (FBS)—induces elastic no-slip boundaries at the medium:FC40 interface. We then develop a semi-analytical model that results in a simple power law; it enables the prediction of flow fields and shear stresses throughout a conduit. Finally, we experimentally confirm the accuracy of the model applied to single conduits and complex vascular trees.

## Results

### Circuit fabrication and operation

**Fig. 1:**
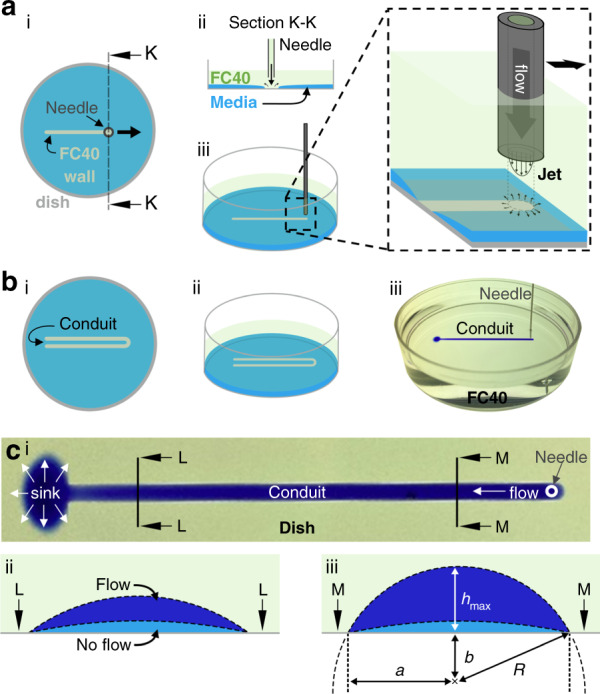
Workflow. **a** Building one straight fluid wall by jetting. (i) After wetting a virgin dish with medium and overlaying immiscible FC40, we lower a dispensing needle until it is just above the medium, where it jets FC40 onto the bottom. As the needle moves laterally, the submerged jet sweeps medium aside to leave an FC40 wall pinned to the bottom. (ii) 2D view of Section K-K. (iii) 3D side view of jet. **b** Operation of a simple circuit—an open-ended conduit. (i) 2D and (ii) 3D views of the conduit. (iii) A syringe pump drives dye (added to aid visualization) through the conduit; the dye front has just reached the open end of the conduit and is emptying into the rest of the dish (the sink). **c** Conduit geometry. (i) Top view of the conduit without the needle (position indicated). (ii) Changes in the conduit cross-section at L-L in c(i) during flow. Before flow is initiated, the conduit is bounded by FC40 walls shaped like a spherical cap (light blue). During flow, the walls morph due to the increase in pressure, and the conduit height increases (dark blue). (iii) Changes at M-M. Before flow, the cross-section is similar to that at L-L; during flow, *h*_*max*_ increases more than at L-L, as the pressure is higher. R = radius of curvature of the cap. b = distance from the center of the cap to the dish surface. a = conduit half-width.

Microfluidic circuits are fabricated using a microjet.^20^ A thin layer of cell-growth medium (i.e., DMEM) plus 10% fetal bovine serum (FBS) in a virgin Petri dish is overlaid with FC40, and the tip of a needle held by a 3-way traverse (a ‘printer’) is lowered through the FC40 until it is located just above the medium (Fig. 1a). The needle is filled with FC40 and connected to a syringe pump; then, starting the pump jets FC40 through the medium on to the bottom of the dish, and the submerged jet sweeps some medium aside. As FC40 wets polystyrene better than the medium, some fluorocarbons remain stuck to the bottom. Moving the needle above the dish in the desired 2D pattern creates a fluid FC40 wall on the bottom of the dish. We begin with a simple open-ended conduit (Fig. 1bi), where medium is infused through a dispensing tube/needle inserted through the fluid ceiling of the conduit (Fig. 1biii); it flows down the conduit and out through the open end into the rest of the dish (the sink; Fig. 1ci).

### The challenge: predicting the flows when the fluid walls morph

Unlike the solid walls in traditional microfluidic circuits, which have fixed shapes, FC40 walls/ceilings morph during flow above an unchanging footprint. The curvature of confining FC40 walls is determined by interfacial forces and hence can change during flow. As the Bond number is low, the conduits have cross-sections like the segments of circles; the walls are pinned to the surface of the dish along the triple contact-line where the medium, FC40, and polystyrene meet (Fig. 1c). Analogous to horizontal pipe flow, there is a pressure gradient in the flow direction along a conduit, and hence, the fluid walls morph in response to this gradient. The Laplace pressure at any axial position along the conduit is given by ${{{\mathrm{{\Delta}}}}}P_{\rm{conduit}} = \frac{\gamma }{R}$ (where *γ* is the interfacial tension and *R* is the radius of curvature). As Δ*P* is inversely proportional to *R*, a decrease in *P* results in an increase in *R*, which translates into a decrease in conduit height (defined as *h*_*max*_ in Fig. 1ciii). Thus, the conduit height falls from the input end (high pressure) to the open end (low pressure; contrast the dark blue sections in Fig. 1cii and Fig. 1ciii). Our challenge is to develop a semi-analytical solution that enables the prediction of velocity and shear stress distributions throughout such conduits.

### Measuring the conduit height

**Fig. 2:**
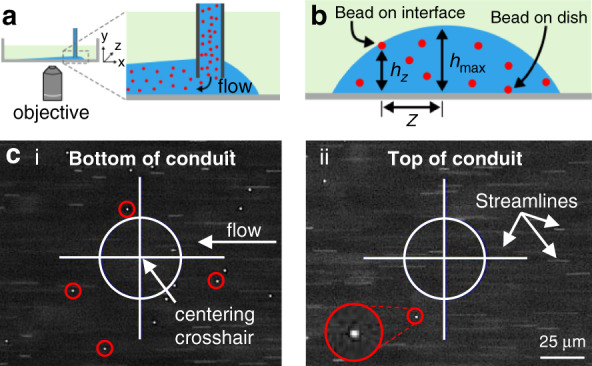
Experimentally measuring conduit heights. **a** Overview. The conduit is imaged under a microscope as medium + red fluorescent beads flow through it. **b** Conduit cross-section. Most beads travel down the conduit, but a few are stuck on the bottom or the ceiling. *h*_*max*_ is calculated after *z* (the distance between a bead stuck on the ceiling and the axial centerline in the crosshairs) and *h*_*z*_ are measured using the geometry of a segment of a circle. **c** Images of beads after focusing on the bottom (i) and (ii) top of the conduit. Stuck and stationary beads appear as dots, and moving beads appear as blurs (‘streamlines’) due to the long exposure.

As the conduit height reflects the local pressure, we first develop a method to measure it. Medium containing red fluorescent beads is perfused through a conduit sitting on an inverted microscope (Fig. 2a); most beads travel steadily through the conduit, but some remain stationary and stuck to the conduit floor (the dish) or ceiling (the medium:FC40 interface; Fig. 2b). In still photographs taken with the focus on the floor or ceiling, the moving majority appear as blurred streamlines due to the long exposure, and the static minority appear as bright in-focus dots (Fig. 2c). After the distance of a stationary bead stuck to the ceiling from the conduit centerline (*z*) is measured, the conduit height (*h*_max_) is calculated using geometry (Fig. 2b) and corrected for refractive effects (Supplementary Information).

### FBS creates a no-slip boundary at the medium:FC40 interfaces

As the conduit floor is solid, the no-slip boundary condition applies there and results in zero velocity at the medium:polystyrene interface; therefore, it is unsurprising that beads on the bottom remain stationary (Fig. 2ci). However, it is surprising that some are stationary at the upper interface (Fig. 2cii); we might anticipate that they would be in motion (the viscosity ratio of FC40:medium is ~4). Testing the medium with and without FBS shows that adding serum induces no-slip conditions. As the constitution of FBS is ill-defined and varies from batch to batch, we replace it with the better-defined KnockOut serum replacement (SR) and find that it has the same effect. It was previously shown that cells attach and grow at the interface between fluorocarbon fluids and tissue culture medium.^24,25^ As with solid surfaces, cells interact with a monolayer of denatured proteins that adsorb to the interface.^24,25^ This monolayer typically consists of serum proteins (in which there is an abundance of albumin); this allow cells to attach and thus presumably induces no-slip conditions in these liquid-liquid interfaces. In our experiments, the medium flows past static FC40, so we change the conditions; FC40 is jetted through FC40 overlaying a static drop containing beads. In the absence of FBS, FC40 flow induces rapid bead motion, as forces are transmitted through the interface; with the addition of FBS to the drop, the beads immediately stop moving (Movie S1). We conclude that FBS and SR (two of the most widely used additives in mammalian cell culture media) rapidly induce the formation of a no-slip boundary. We now apply this boundary condition to develop a theoretical model.

### Semi-analytical model to describe fluid-walled conduits

Our initial aim is to model a straight conduit with known flow rates provided by a syringe pump (Fig. 1c). We first consider the standard solution for flow between parallel plates derived from the momentum equation (the Supplementary Information gives full derivations for this and other equations). The resultant pressure gradient is $\frac{{dP}}{{dx}} = \frac{{8\mu u_{\max}}}{{h_{\max}^2}}$ (*μ* = viscosity, *u*_max_ = maximum velocity). As the cross-section of fluid-walled conduits can be represented by the segment of a circle where height is expressed locally as $h_z = \sqrt {R^2 - z^2} - b$, the flow rate (*Q*) becomes ($Q = uA$; *u* = velocity, *A* = cross-sectional area):

1 $$\begin{array}{*{20}{c}} {Q = \frac{4}{3}\mathop {\int }\limits_0^a \frac{{h_z^3u_{\max}}}{{h_{\max}^2}}dz = \frac{4}{3}\frac{{u_{\max}}}{{h_{\max}^2}}\mathop {\int }\limits_0^a \left( {\sqrt {R^2 - z^2} - b} \right)^3dz} \end{array}$$

The integrand is rearranged and nondimensionalized using $\eta = \frac{z}{a}$ and evaluated for the condition $a \gg h_{\max}$ (defined as $\frac{{h_{\max}}}{a} \le 0.2$, Fig. S5), which—critically for the following solution—yields an approximately constant value for the integral, giving $Q = 0.61\;u_{\max}a\;h_{\max}$. Substituting this into the pressure gradient for flow between two parallel plates, we obtain $\frac{{dP}}{{dx}} = \frac{{13.04\mu Q}}{{a\;h_{\max}^3}}$. For $a \gg h_{\max}$, $R = \frac{{a^2}}{{2h}}$, which combined with ${{{\mathrm{{\Delta}}}}}P_{\rm{conduit}} = \frac{\gamma }{R}$ yields the semi-analytical expression for conduit height $h_{\max}(x) = ( {\frac{{26.08Q\mu ax}}{\gamma } + h_0^4} )^{0.25}$ (*h*_0_ is the maximum height at the exit, where $x = 0$). For a conduit height-to-width ratio $\frac{{h_{\max}}}{a} = 0.2$ (within our operational range), the associated error is ~4%, which is a result of our simplification of the radius of curvature of a conduit from $a \gg h_{\max}$.

This solution enables the height at any position in a conduit to be predicted if the boundary condition *h*_0_ is known. Evaluating the magnitude of the bracketed term $\frac{{26.08Q\mu ax}}{{\gamma h_0^4}},$ we find that for a conduit with *a* = 400 µm, *μ* = 1 cP, *Q* = 25 µL/h, *γ* = 0.04 N/m, and *h*_0_ = 20 µm (as an order of magnitude reference), the effect of the boundary condition on the height >1 mm away from the exit is small. This provides a favorable corollary; height can be predicted by the simplified power law:

2 $$\begin{array}{*{20}{c}} {h_{\max}(x) = \left( {\frac{{26.08Q\mu ax}}{\gamma }} \right)^{0.25}} \end{array}$$

Hence, we do not require *h*_0_ to be known to predict the conduit height for most experimental conditions of interest.

### The semi-analytical solution predicts experimentally measured heights

**Fig. 3:**
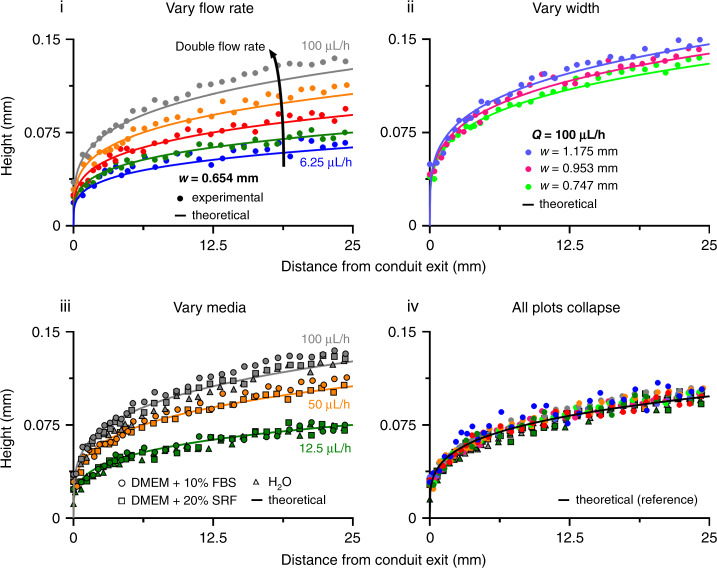
Effects of varying conditions on the maximum conduit height. Individual points: *h*_max_ measured as in Fig. 2 over the conduit length (25 mm). Continuous lines: theoretical predictions based on Eq. 2 (in iv, using *Q* = 25 µL/h and *w* = 953 µm). (i) Varying flow rate (conduit width ~650 µm). (ii) Varying conduit width (flow rate 100 µL/h). (iii) Varying medium flowing through the conduit (width ~650 µm). Conduits are either prepared using DMEM + 10% FBS and perfused with the same medium (circular points) or H_2_O (triangular points) or prepared and perfused using DMEM + 20% SR (square points). The heights at each flow rate are almost identical to those of DMEM + 10% FBS. (iv) Height measurements of all conduits collapsed onto a single reference plot according to Eq. S38 (color codes as in i and ii), demonstrating that Eq. 2 predicts the conduit heights for varying conditions.

We next measure the heights of conduits made with DMEM + 10% FBS (from the exit to 25 mm upstream) using the approach outlined in Fig. 2 and compare the results with those predicted by Eq. 2 using *γ* = 0.022 N/m (obtained from pendant-drop tensiometry; see the Supplementary Information) and *μ* = 0.94 cP (at 25 °C;^24^). In all cases, the predicted results fit the experimental data well, for example, as the flow and conduit width vary (6.25 µL/h ≤ *Q* ≤ 100 µL/h in Fig. 3i and ~750–1175 µm in Fig. 3ii). Good fits are also obtained with conduits printed and perfused with medium plus 20% SR and conduits printed with medium plus 10% FBS and then perfused with water (Fig. 3iii); this suggests that components in FBS and SR are deposited on the conduit walls and ceilings during conduit construction and create long-lived no-slip boundaries. Therefore, we examine the stability of the boundary with and without continuous flow for 24 h before measuring the heights (with flow). Although the variance in the height over time is small between experiments (Fig. S8), the dynamic nature of interfacial tension can be expected to introduce a time-dependent variable explaining the small differences measured. We also show that the measured heights match the predictions through mapping on a single reference plot (Fig. 3iv; Eq. S42). Finally, we compare the predictions obtained from the semi-analytical solution with those from a numerical model that does not require the *h*/*a* simplification implicit in our power law; there is excellent agreement at scales relevant to this study (Fig. S6; Supplementary Information).

### Predicting the wall shear stress

The wall shear stress, *τ*_max_, is evaluated at $y = \pm \frac{h}{2}$, with $\tau _{\max} = \mu \frac{{\partial u}}{{\partial y}}$ and $\frac{{\partial u}}{{\partial y}} = \frac{{8yu_{\max}}}{{h_{\max}^2}}$. We then use the relationship $u_{\max(z)} = \frac{{u_{\max(0)}}}{{h_{\max}^2}}h_z^2$ to infer $\tau _{\max(z)} = \tau _{\max(0)}\frac{{h_z}}{{h_{\max}}}$. *τ*_max_ is then expressed in terms of *h*_max_ using Eq. 2, yielding:

3 $$\begin{array}{*{20}{c}} {\tau _{\max(z)} = 1.28\sqrt {\frac{{\mu \gamma Q}}{{a^3x}}} \frac{{h_z}}{{h_{\max}}}} \end{array}$$

### Chokes reduce height variations down conduits

**Fig. 4:**
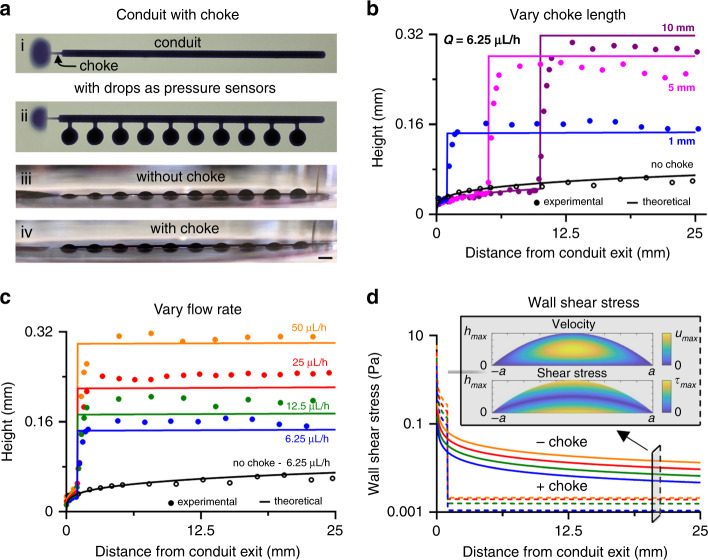
Chokes suppress variations in *h*_*max*_ and shear stress along conduits. Generally, conduits (width ~950 µm) are connected to exit chokes (width ~400 µm). The lines in the graphs show the theoretical results from Eq. 2. **a** Images. Blue dye is added to aid visualization of the flow to the left. Bar: 2 mm. (i) Top view of conduit (length 24 mm) with choke (length 1 mm). (ii) Top view of a conduit + choke (as i) with attached pressure sensors/drops. (iii, iv) Side views of a conduit (length 25 mm) + sensors and conduit (length 24 mm) + sensors plus choke (1 mm). The sensor-drop height reflects the local pressure in the main conduit. Without a choke, the drop height decreases in the direction of flow with distance from the exit; with a choke, the heights (and thus the pressures) remain more uniform down the conduit. **b** Varying choke lengths (1, 5, and 10 mm; as the choke length increases, the conduit length decreases, so all structures are 25 mm long). **c** Height variations are stabilized at different levels at different flow rates (conduit length 24 mm, choke length 1 mm). **d** Shear stresses (calculated semi-analytically from Eq. 3) down conduits ± chokes (conduit length 24 mm, choke length 1 mm). The line colors represent the same conditions as those in panel (**c**). Solid lines—without choke; dashed lines—with choke. Chokes ensure that the shear stress is more uniform down the conduit. Inset: normalized velocity and shear stress profiles applied to any flow rate across the circular cross-section of any conduit ± choke (see Supplementary Information).

Controlling shear stress is essential for many cell studies; this is easily achieved in solid-walled systems with fixed geometries.^12^ In our conduits, the height increases from the exit as one progresses further upstream, which translates into corresponding variations in shear stress. In addition, $\frac{{dh}}{{dx}}$ progressively decreases as the distance from the exit increases (from Eq. 2, $h \propto x^{0.25}$, and so $\frac{{dh}}{{dx}} \propto x^{ - 0.75}$). Consequently, approximately uniform fields of wall shear stress ($\frac{{d\tau }}{{dx}}$) are only found tens of centimeters from the exit, so conduits with the relatively uniform fields required by biologists cannot be built in a 6 cm dish. Therefore, we add a short narrow conduit (a choke) to the exit of the conduit so that the choke bears most of the pressure drop and creates a field of nearly uniform shear throughout the conduit (Fig. 4ai). Due to the choke, $h_{\rm{conduit}} \propto ( {\frac{{a_{\rm{conduit}}}}{{a_{\rm{choke}}}}} )^2$; then, the effective length (*x*_eff_) introduced by the choke, at which $h = h_{\rm{conduit}}$ for a conduit without a choke, is $x_{\rm{eff}} = x_{\rm{choke}}( {\frac{{a_{\rm{conduit}}}}{{a_{\rm{choke}}}}} )^7$. From Eq. 3,$( {\frac{{d\tau }}{{dx}}} )_{{\rm{no}}\;{\rm{choke}}} \propto ( {\frac{1}{{ax}}} )_{{\rm{conduit}}}^{\frac{3}{2}}$, so $( {\frac{{d\tau }}{{dx}}} )_{\rm{choke}} \propto ( {\frac{1}{{a_{\rm{conduit}}x_{\rm{eff}}}}} )^{\frac{3}{2}}$. Hence, the change in $\frac{{d\tau }}{{dx}}$ from the addition of a choke is $\frac{{( {\frac{{d\tau }}{{dx}}} )_{{\rm{no}}\;{\rm{choke}}}}}{{( {\frac{{d\tau }}{{dx}}} )_{{\rm{choke}}}}} \propto ( {\frac{{a_{\rm{conduit}}}}{{a_{{\rm{choke}}}}}} )^{\frac{{21}}{2}}$. Therefore, for conduits in Fig. 4b, the change in *τ* over 1 cm upstream of the choke is marginal (in order of increasing choke length, the values are 0.85%, 0.20%, and 0.15%), and the wall shear stress is effectively constant in the flow direction.

To illustrate the effect of a choke on the conduit height, we print a conduit with 10 connected drops (Fig. 4aii). During flow, the pressure in each drop matches the local pressure in the conduit at the connection point; therefore, each drop serves as an independent pressure sensor. Without a choke, the drop height decreases in the direction of flow as the pressure falls (Fig. 4aiii). With a choke, all sensors have approximately similar heights (Fig. 4aiv). The wall shear stresses calculated from Eq. 3 also show that choked conduits have more constant fields along their lengths that are up to 2 orders of magnitude smaller than those of choke-free conduits (Fig. 4d). The predicted velocity and shear-stress fields across the conduit width are shown in the inset in Fig. 4d. Strikingly, 25% of the width experiences < 5% variation in shear stress (yellow region in the inset). Human umbilical vein endothelial cells (HUVECs)—chosen because they are known to respond to shear stress—grow as expected^5^ in such a choked conduit (Fig. S12).

### Predicting flows in complex networks

This theory for single conduits can be extended to networks where conduits split or merge (as in vascular trees). We begin with a network in which input (parent) and output (daughter) conduits have the same width. The output flow (*Q*_*d*_) is determined by continuity, so for one input (flow rate *Q*_*p*_) splitting into *n* outputs, $Q_d = \frac{{Q_p}}{n}$. If instead, *m* inputs (with the same *Q*_*p*_) merge into a single output, then *Q*_*d*_ = *m* × *Q*_*p*_. This process can be repeated to determine flows emerging from each node in a complex tree. The semi-analytical solution is then applied iteratively through each conduit starting from the exit.

**Fig. 5:**
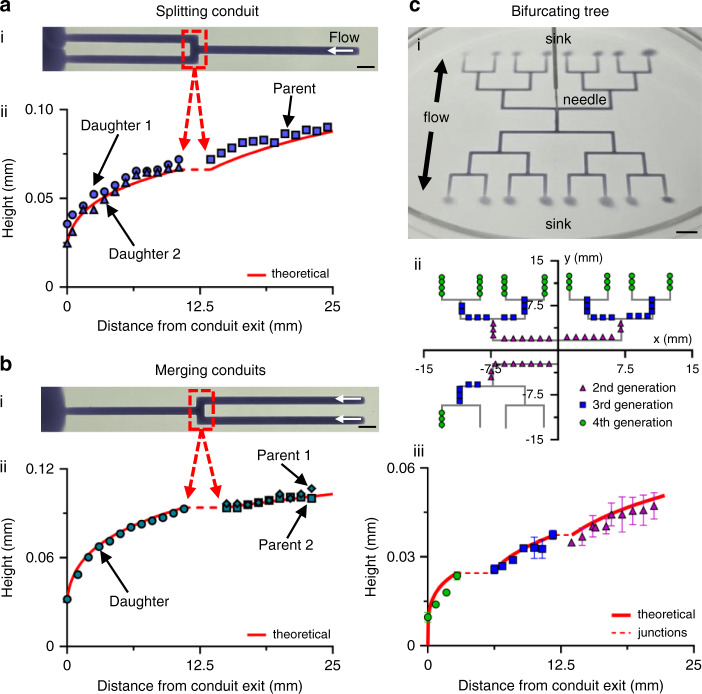
Splitting, merging and bifurcating circuits. Conduit heights are measured as described in Fig. 2. Red line in plots: theoretical height predictions from Eq. 2 (dashed portions correspond to junctions and assume no local pressure drop). Bars in images: 2 mm. **a** Splitting conduit. (i) Image (conduit width ~650 µm). (ii) Heights down parent and daughters (input into parent 25 µL/h). **b** Merging conduits. (i) Image (conduit width ~650 µm). (ii) Heights down conduits (both parents perfuse at 25 µL/h). **c** Bifurcating tree. (i) Image (6 cm dish; conduit width ~550 µm). (ii) Tree plan (gray line) showing where heights are measured (colored symbols indicate the daughter generation). (iii) Plot indicating the concordance between the measured (symbols colored the same as those in cii) and predicted heights.

We first study one conduit splitting into two daughters (Fig. 5a), then the same structure with reversed flow (Fig. 5b), and finally a bifurcating tree with four generations of daughters (Fig. 5c). All parents and daughters have the same widths, but the flow area at the junction increases, so the local pressure drop is small and assumed to be negligible. The theoretical predictions fit well with the experimental height measurements (Fig. 5). This theory may also be extended to networks in which conduits have different lengths and widths (Supplementary Information).

## Discussion

Our challenge was to predict flow through a straight fluid-walled conduit built using a cell culture medium on a standard 6 cm Petri dish; during flow, the cross-section of such a conduit inevitably morphs above an unchanging footprint (i.e., it expands as flow increases and shrinks with distance from the input; Fig. 1c). Our approach is based on the recognition that the conduit height reflects the local pressure. Therefore, we develop a method to measure height (Fig. 2) and find that the addition of FBS or SR to the medium creates a solid medium:FC40 interface that induces no-slip boundary conditions; this defines the boundary conditions for our model (Fig. 2). Therefore, it is likely that fluids rich in albumin (e.g., that in the vasculature, lymph, cerebrospinal fluid, and vitreous humor) yield the same no-slip conditions. We next establish a simple power law with a fixed exponent (Eq. 2) by approximating the conduit cross-section and applying no-slip boundary conditions. This power law enables the prediction of the conduit height, flow field, and shear stress (Eq. 3) anywhere in a conduit; it requires no additional boundary conditions beyond the conduit footprint, flow rate, viscosity of the medium, and interfacial tension. This level of information is essentially similar to that required with laminar flow through a solid-walled pipe, except the pipe diameter now morphs in the flow direction to enable the pressure gradient induced by interfacial tension to satisfy the momentum equation. This law also agrees with numerical predictions for conduits satisfying *h*/*a* < ~0.2, which is in the practical range. This enables users to design conduits and to predict a priori flows and shear stresses matching their specific requirements, including those with essentially uniform shear-stress profiles through the addition of chokes (in the absence of chokes, such conduits would be too long to fit in a 6 cm dish). We also experimentally demonstrate the interplay between the conduit height and pressure by printing 10 drops connected at different points down a conduit; then, during flow, each drop serves as an independent pressure sensor (as the height reflects the local pressure; Fig. 4a). We also experimentally verify the accuracy of the model by varying flows through conduits with a range of widths (Fig. 3), splitting and merging conduits (Fig. 5a, b) and using a fractal vascular tree (Fig. 5c). As this form of open microfluidics offers multiple advantages over traditional closed systems^20–22^ we anticipate that this model will increase the uptake of these circuits in domains benefiting from flow (e.g., when generating chemotactic gradients using laminar streams) and well-defined shear stress (e.g., when studying thromboses in vascular trees).

## Materials and methods

### Fluid-walled microfluidic circuit fabrication

All circuits were fabricated using a custom-made programmable printer (iotaSciences Ltd, Oxford, UK). The printer is fitted with a syringe pump that drives a 1 mL glass syringe (Hamilton, Reno, Nevada, USA). The syringe is filled with the immiscible and bioinert fluorocarbon FC40 (FC40STAR^®^, iotaSciences Ltd, Oxford, UK) and connected via polytetrafluoroethylene (PTFE) tubing (26 G, Adhesive Dispensing Ltd, Milton Keynes, UK) to a laser-cut jetting needle (25 G, 70 µm inner diameter, Oxford Lasers, Didcot, UK) held by the printer’s 3-axis traverse system.

Circuits were made on 60 mm tissue culture-treated dishes (Corning, 430166). First, 1 mL of cell culture medium is pipetted on the dish and swirled until it covers the entire bottom of the dish. The medium is either DMEM (Merck) + 10% FBS (Merck) or DMEM + 20% SR (Thermo Fisher). The dish is then tilted so that the excess medium drains to the side, and most of the medium removed by pipetting, leaving a thin residual film (~30 µm thick) on the bottom. This film is overlaid with 2 mL FC40 (Fig. 1a), the dish is placed on the stage of the printer, and the jetting needle is lowered until the tip is 0.5 mm above the bottom of the dish. FC40 is now jetted (8 ﻿µL/s) as the needle moves laterally above the surface of the dish. As the submerged FC40 jet contacts the dish’s surface, it pushes the medium aside to leave fluid walls of FC40 pinned to the dish by interfacial forces. These walls then shape the conduit.

### Perfusing fluid-walled conduits

A 250 µL glass syringe (Hamilton) filled with medium and placed in a syringe pump (PhD Ultra, Harvard Apparatus) is connected via PTFE tubing to a 25 G stainless-steel hollow needle (Adhesive Dispensing Ltd). The needle is lowered through the ceiling into the conduit ~30 mm from the exit until it is ~100 µm above the bottom of the dish, and the dish is placed on the stage of an inverted microscope. The syringe pump is then started, and conduit heights are measured once the pressures in the conduits reach equilibrium, after ~30 min. The open-ended straight conduits are 25 mm long unless stated otherwise.

### Measuring the conduit height

The conduits in Figs. 3 and 4 were imaged on a Zeiss Axio Observer inverted microscope with a 10X Plan Apochromat Air objective (NA = 0.3), and those in Fig. 5 and S8 were imaged on an Olympus IX53 inverted microscope with a 40X objective (NA = 0.55). The conduit heights are measured as the conduits are perfused with medium plus red fluorescent beads (1:1000 mixture; 0.5 µm diameter; FluoSpheres, F8812, Thermo Fisher). After equilibration for ~30 min after the beginning of perfusion, some beads remain stationary on the surface of the dish or the medium:FC40 interface and are used for height measurements (Fig. 2). To validate the theoretical model, we experimentally measure *h*_max_ and correct the heights for the apparent depth due to refraction (Eq. S40).

The Zeiss microscope has a motorized stage and a digital controller that can record coordinates to within tens of nm. It displays real-time images on a monitor, with cross-hairs indicating the exact position (Fig. 2c). Starting from the conduit exit, this digital controller is used to position the objective close to the center of the conduit, and a static bead on the medium:FC40 ceiling is brought into focus. The coordinates are recorded, and the stage is lowered to focus on a static bead on the dish surface (there are typically many). The coordinates are again recorded. The stage is then moved laterally to record the edge coordinates of the conduit. The offset *z* between the bead on the ceiling and the center of the conduit and the corresponding height at this point, *h*_*z*_, are now calculated. *h*_max_ is then inferred using trigonometry, and the measurements are repeated along the length of the conduit. In the first 5 mm (where the conduit height increases most rapidly), measurements are taken every 0.5 mm, and in the remaining 20 mm steps, the measurement interval is increased to 1 mm. The stage of the Olympus microscope lacks digital control and is less precise, and the approximate location of the center of the conduit is determined using the circular nature of the fluid interface; moving the objective upwards scans through beads stuck on the interface, getting closer to the top of the conduit as the objective is raised. The top is identified when only one or a few beads remain in focus (typically in an axial strip), and this position is recorded using the scale on the objective knob of the microscope (ticks spaced in 1 µm increments). The position on the surface of the dish directly below this bead is also recorded, and the difference is *h*_max_. This process is then repeated along the entire conduit as before. Pictures of the conduit are then taken with a 4X objective to obtain its average width.

Due to mismatches between the refractive indices of the culture medium and air, height differences measured on microscopes using the focal position of beads do not reflect the axial movement of the microscope objective; rather, the height differences appear smaller.^26^ Height corrections are dependent on the objective numerical aperture and refractive indices, which for our experiments are found to be Δ*f* = *α* × Δ*s*, where Δ*f* is the actual conduit height, Δ*s* is the measured height, and *α* is a constant (*α* = 1.36 for Zeiss measurements; *α* = 1.45 for Olympus measurements).

## Supplementary information

Supplementary Information

Movie S1
