# Supplementary material for: Predicting flows through microfluidic circuits with fluid walls
Source: Microsyst Nanoeng. 2021 Nov 18;7:93. doi: 10.1038/s41378-021-00322-6 (PMC8599700; doi:10.1038/s41378-021-00322-6)
Supplement: Supplementary file 1 — Supplementary Information [file 41378_2021_322_MOESM1_ESM.docx]

**Supplementary Information for**

**Predicting flows through microfluidic circuits with fluid walls**

**Cyril Deroy, Nicholas Stovall-Kurtz, Federico Nebuloni, Cristian Soitu, Peter R. Cook*****, Edmond J. Walsh***

Corresponding authors:

*Peter R. Cook

**Email:**  [peter.cook@path.ox.ac.uk](mailto:peter.cook@path.ox.ac.uk)

*Edmond J. Walsh

**Email:**  [edmond.walsh@eng.ox.ac.uk](mailto:edmond.walsh@eng.ox.ac.uk)

**This Word file includes:**

Supplementary text

Figures S1 to S12

Tables S1 to S4

Legend for Movie S1

SI References

**Other supplementary materials for this manuscript include the following:**

Movie S1

Supplementary Information Text

**Semi-analytical solution for fluid-walled conduits**

The solution to flow through fluid-walled conduits is derived from the simplified Navier-Stokes equation:

$$\begin{aligned} \rho\left( u\frac{\partial u}{\partial x}+v\frac{\partial u}{\partial y} \right)=-\frac{\partial P}{\partial x}+\mu\left( \frac{\partial^{2}u}{\partial x^{2}}+ \frac{\partial^{2}u}{\partial y^{2}} \right)\#\left( S1 \right) \end{aligned}$$

Flow is laminar (the Reynolds number of our conduits never exceeds 0.1; Table S1), unidirectional ($v=0$), and fully-developed ($\frac{\partial u}{\partial x}=\frac{\partial^{2}u}{\partial x^{2}}=0$). The simplified equation becomes:

$$\begin{aligned} \frac{\partial P}{\partial x}=\mu\frac{\partial^{2}u}{\partial y^{2}}\#\left( S2 \right) \end{aligned}$$

From Fig. 1C, we determine these geometrical relationships:

$$\begin{aligned} a^{2}+b^{2}=R^{2} \end{aligned}$$

$$\begin{aligned} R=b+h_{max} \end{aligned}$$

$$R=\frac{a^{2}+h_{max}^{2}}{2h_{max}} b=\frac{a^{2}-h_{max}^{2}}{2h_{max}}$$

Conduit height, $h_{z}$, at any location across the half width (Fig. 2B) is:

$$\begin{aligned} h_{z}=\sqrt{R^{2}-z^{2}}-b\#\left( S3 \right) \end{aligned}$$

At the center of the conduit ($z=0$), height is maximal and corresponds to $h_{max}$; at pinning lines ($z=\pm a$), it is 0. The width and length of the conduit are set by the user and do not change over time. However, $h_{max}$ depends on the pressure which changes during flow as fluid walls morph; it increases with increasing pressure, and decreases in the direction of flow. From the provided boundary conditions, the dimensionless velocity profile is assumed to be the same as that used in the classical solution of Poiseuille flow between parallel plates, and – as media:FC40 interfaces act as solid boundaries – we assume the no-slip boundary condition applies:

$$@y=\pm\frac{h_{max}}{2};u=0 and @y=0; \frac{du}{dy}=0$$

Integrating Eq. S2 twice yields:

$$\frac{du}{dy}=\frac{1}{\mu}\frac{dP}{dx}y+c_{1}, u\left( y \right)=\frac{1}{2\mu}\frac{dP}{dx}y^{2}+c_{1}y+c_{2}$$

Applying boundary conditions, we find:

$$c_{1}=0; c_{2}=-\frac{1}{8\mu}\frac{dP}{dx}h_{max}^{2}$$

And so $u(y)$ can be expressed as:

$$\begin{aligned} u\left( y \right)=\frac{1}{2\mu}\frac{dP}{dx}\left( y^{2}-\frac{h_{max}^{2}}{4} \right)\#\left( S4 \right) \end{aligned}$$

Since $u\left( y \right)=u_{max} @ y=0$, the flow’s maximum velocity becomes:

$$\begin{aligned} u_{max}=-\frac{h_{max}^{2}}{8\mu}\frac{dP}{dx}\#\left( S5 \right) \end{aligned}$$

$$\begin{aligned} \therefore u\left( y \right)=\left( 1-\frac{4y^{2}}{h_{max}^{2}} \right)u_{max}, \frac{dP}{dx}\propto\frac{u_{max}}{h_{max}^{2}} \end{aligned}$$

Across the conduit’s width (in the z-direction), $\frac{dP}{dx}$ = constant; hence, the relationship between the maximum flow velocity across a width, $u_{max(z)}$, to the maximum conduit velocity, $u_{max(0)}$, is:

$$\frac{u_{max(0)}}{h_{max}^{2}}=\frac{u_{max(z)}}{h_{z}^{2}}$$

$$\begin{aligned} u_{max(z)}= \frac{u_{max(0)}}{h_{max}^{2}}h_{z}^{2}\#\left( S6 \right) \end{aligned}$$

The total flow rate through the conduit is found by integrating the velocity profile:

$$Q=\int_{-a}^{a} \int_{-\frac{h_{z}}{2}}^{\frac{h_{z}}{2}} u\left( y \right)dydz$$

Evaluating the first integral provides flow rate per unit length as:

$$Q^{'}=\int_{-\frac{h_{z}}{2}}^{\frac{h_{z}}{2}} u\left( y \right)dy=-\frac{h_{z}^{3}}{12\mu}\frac{dP}{dx}=\frac{2}{3}u_{max(z)}h_{z}$$

$$\begin{aligned} \therefore Q=\int_{-a}^{a} \frac{2}{3}u_{max(z)}h_{z}dz\#\left( S7 \right) \end{aligned}$$

Substituting for $u_{z}$ in Eq. S7 as in Eq. S6 yields:

$$Q=\frac{2}{3}\frac{u_{max(0)}}{h_{max}^{2}}\int_{-a}^{a} h_{z}^{3}dz$$

And since from geometry $h_{z}=\sqrt{R^{2}-z^{2}}-b$:

$$\begin{aligned} \therefore Q=\frac{4}{3}\frac{u_{max(0)}}{h_{max}^{2}}\int_{0}^{a} \left( \sqrt{R^{2}-z^{2}}-b \right)^{3}dz\#\left( S8 \right) \end{aligned}$$

The integrand is then normalized using $\eta=\frac{z}{a}$ and evaluated over $\frac{a}{h_{max}}$:

$$\begin{aligned} Q=\frac{4}{3}h_{max}u_{max(0)}a\int_{0}^{1} \frac{\left( \sqrt{R^{2}-\eta^{2}a^{2}}-b \right)^{3}}{h_{max}^{3}}d\eta\#\left( S9 \right) \end{aligned}$$

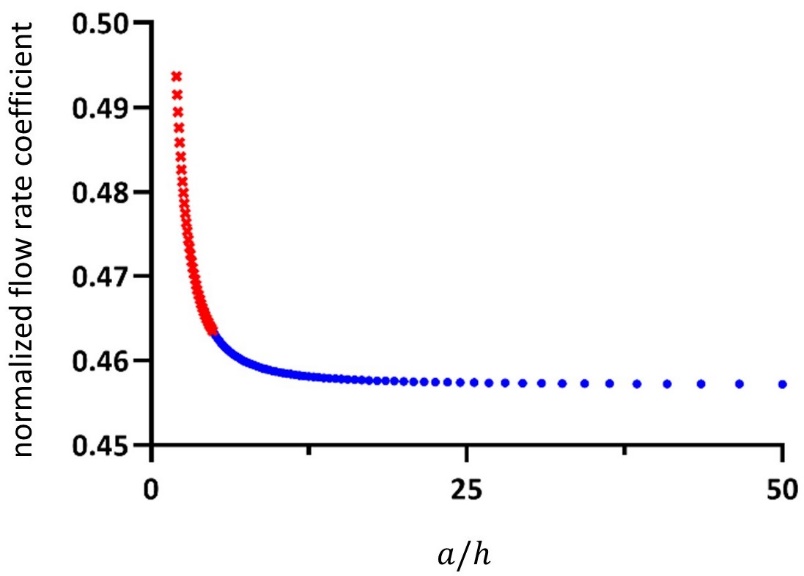


**Fig. S1. Normalized volumetric flow rate coefficients for a range of conduit half width-to-height ratios.** The integrand in Eq. S9 is evaluated for $0<a/h\leq50$. Ratios less than 5 (red crosses, $a\sim h$) are ignored. Ratios greater than 5 (blue dots, $a\gg h$) correspond to conduit geometries typically observed in this study. The average normalized flow rate coefficient in this region corresponds to 0.46.

For heights typically observed here ($a\gg h_{max}$; Fig S1), flow rate is:

$$Q=\frac{4}{3}h_{max}u_{max(0)}a(0.46)$$

$$\begin{aligned} \therefore u_{max(0)}=\frac{Q}{0.61h_{max}a}\#\left( S10 \right) \end{aligned}$$

Now substituting the relationship for $u_{max(0)}$ into Eq. S5 yields:

$$\begin{aligned} h_{max}^{3}\frac{dP}{dx} =\frac{13.11Q\mu}{a}\#(S11) \end{aligned}$$

The radius of curvature of the conduit is defined as $R=\frac{{a^{2}+h}_{max}^{2}}{2h_{max}}$, however when $a\gg h_{max}$ the radius of curvature can be simplified as:

$$\begin{aligned} R=\frac{a^{2}}{2h_{max}}\left( 1+\frac{h_{max}^{2}}{a^{2}} \right)\approx\frac{a^{2}}{2h_{max}}\#\left( S12 \right) \end{aligned}$$

Since change in hydrostatic pressure is assumed to be negligible along the conduit, the local pressure at any x-location can be characterized by the local Laplace pressure in accordance with the simplified Young-Laplace equation for interfaces represented as the arc of a circle (${\Delta P}_{conduit}=\frac{\gamma}{R}$). Using the simplified cross-sectional radii of curvature expressed in Eq. S12 produces the following equation defining pressure increase across the interface:

$$\begin{aligned} \Delta P_{interface}=\frac{\gamma}{R}\approx\gamma\left( \frac{2h_{max}}{a^{2}} \right) \end{aligned}$$

$$\begin{aligned} \therefore\frac{dP}{dx}=\frac{2\gamma}{a^{2}}\frac{dh}{dx}\#\left( S13 \right) \end{aligned}$$

Substituting Eq. S13 into Eq. S11:

$$\begin{aligned} \frac{2\gamma}{a^{2}}h_{max}^{3}dh=\frac{13.11Q\mu}{a}dx\#\left( S14 \right) \end{aligned}$$

$$\begin{aligned} \therefore h_{max}^{3}dh=\frac{6.55Q\mu a}{\gamma}dx\#\left( S15 \right) \end{aligned}$$

Integrating Eq. S15 then gives:

$$\begin{aligned} h_{max}^{4}=\frac{26.08Q\mu ax}{\gamma}+c_{1}\#\left( S16 \right) \end{aligned}$$

Since at the exit, $h_{max}=h_{0}$, then $c_{1}=h_{0}^{4}$. Hence the semi-analytical solution for predicting conduit center-heights becomes:

$$\begin{aligned} h_{max}(x)=\left( \frac{26.08Q\mu ax}{\gamma}+h_{0}^{4} \right)^{0.25}\#\left( S17 \right) \end{aligned}$$

Reintroducing $P\approx\gamma\left( \frac{2h_{max}}{a^{2}} \right)$ into Eq. S17 solves for pressure as:

$$\begin{aligned} P_{x}=\left( \frac{417.28Q\mu\gamma^{3}x}{a^{7}}+P_{0}^{4} \right)^{0.25}\#\left( S18 \right) \end{aligned}$$

**Importance of conduit exit-height**

Eq. S17 requires an experimental measurement, conduit exit-height, $h_{0}$, to predict the height elsewhere in the conduit. However, consider the relative magnitude of the terms:

$$\frac{26.08Q\mu ax}{\gamma}, h_{0}^{4}$$

When $\frac{26.08Q\mu ax}{\gamma}\gg h_{0}^{4}$, exit height has a negligible effect on conduit height upstream. For given fluids, flow rate, and conduit width, the location along the conduit at which these two terms become equivalent is:

$$\begin{aligned} x=\frac{h_{0}^{4}\gamma}{26.08Q\mu a}\#\left( S19 \right) \end{aligned}$$

For average flow rates and conduit widths used here ($Q=25$ µL/h, $a=0.5$ mm), $x\approx92$ µm. At a conservative distance from the exit (i.e., an order of magnitude greater than $x$; 1 mm) height predictions converge across a range of exit heights (Fig. S2). Hence, a simplified semi-analytical solution requiring no experimental measurements can predict conduit height accurately away from the exit:

$$\begin{aligned} h_{max}(x)=\left( \frac{26.08Q\mu ax}{\gamma} \right)^{0.25}\#\left( S20 \right) \end{aligned}$$

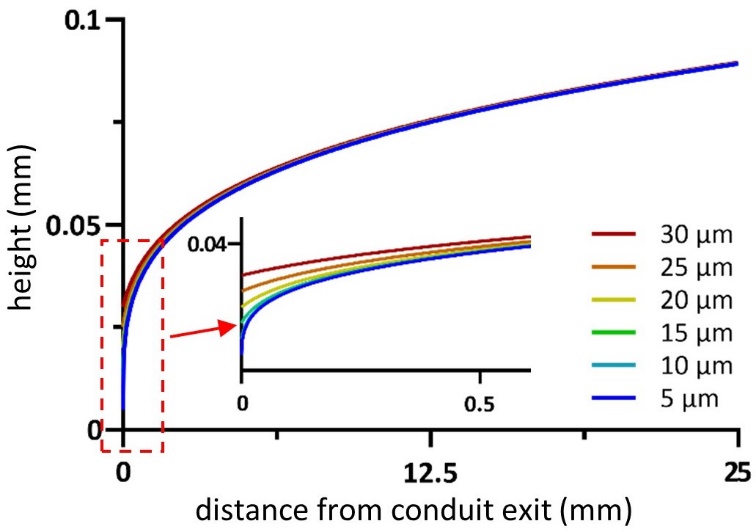


**Fig. S2. The effect of exit height on conduit height is negligible.** Conduit height ($h_{max}$) is calculated using Eq. S17 assuming exit heights ($h_{0}$) of 5 – 30 µm ($Q=25$ µL/h, $a=0.5$ mm). Inset: results for the first 0.6 mm. For low exit heights ($5<h_{0}<20$), conduit heights differ markedly only in the first ~0.1 mm before converging; for larger exit heights ($h_{0}=25 \mathrm{or} 30$ µm) convergence occurs later.

**Numerical solution**

The semi-analytical solution provides a simple powerful method to determine flows. However, it relies on geometrical assumptions (i.e., when deriving the normalized volumetric flow rate constant of Eq. S10, and $R=\frac{a^{2}}{2h}$). Therefore, we derived a numerical solution without these simplifications using the forward Euler method. Pressure along a conduit is solved iteratively in steps of $\Delta x$ as:

$$P^{'}\left( x \right)=\frac{P\left( x+\Delta x \right)-P(x)}{\Delta x}$$

$$\begin{aligned} \therefore P\left( x+\Delta x \right)=P\left( x \right)+\Delta xP^{'}\left( x \right)\#\left( S21 \right) \end{aligned}$$

From Eq. S8, volumetric flow rate is defined as:

$$\begin{aligned} Q=\frac{4}{3}\frac{u_{max}\left( x \right)}{h_{max}^{2}\left( x \right)}\int_{0}^{a} \left( \sqrt{R^{2}-z^{2}}-b \right)^{3}dz\#\left( S22 \right) \end{aligned}$$

The integrand in Eq. S22 is a positive constant dependent only on local cross-section, represented by $\beta$:

$$\begin{aligned} Q=\frac{4}{3}\frac{u_{max}(x)}{h_{max}^{2}(x)}\beta(x)\#\left( S23 \right) \end{aligned}$$

Rearranging for $u_{max}$:

$$\begin{aligned} u_{max}(x)=\frac{3}{4}\frac{h_{max}^{2}(x)}{\beta(x)}Q\#\left( S24 \right) \end{aligned}$$

Given the initial value problem $P^{'}\left( x \right)=\frac{8\mu u_{max}}{h_{max}^{2}}$ (Eq. S5), $P^{'}\left( 0 \right)=\frac{8\mu u_{0}}{h_{0}^{2}}$, and $P(0)=\frac{\gamma}{R}=\frac{2\gamma h_{0}}{a^{2}+h_{0}^{2}}$, Eq. S21 becomes:

$$\begin{aligned} P\left( \Delta x \right)=P\left( 0 \right)+\Delta xP'(0)\#\left( S25 \right) \end{aligned}$$

$$\begin{aligned} P\left( \Delta x \right)=\frac{2\gamma h_{0}}{a^{2}+h_{0}^{2}}+\frac{8\mu u_{0}}{h_{0}^{2}}\Delta x\#\left( S26 \right) \end{aligned}$$

From Eq. S24, $u_{0}=\frac{3}{4}\frac{h_{0}^{2}}{\beta\left( 0 \right)}Q$, hence:

$$\begin{aligned} P\left( \Delta x \right)=\frac{2\gamma h_{0}}{a^{2}+h_{0}^{2}}+\frac{6\mu Q}{\beta\left( 0 \right)}\Delta x\#\left( S27 \right) \end{aligned}$$

Rearranging the Young-Laplace equation yields the radius of curvature at $\Delta x$:

$$\begin{aligned} R(\Delta x)=\frac{\gamma}{P(\Delta x)}\#\left( S28 \right) \end{aligned}$$

This iteration is repeated for $n$ steps $\left( n=\left\{ 1,\ldots,L/\Delta x-1 \right\} \right)$ along any conduit of length $L$, and is valid for as long as the half-width is greater than or equal to the local maximum height ($a>h_{max}$). If this height exceeds the half-width, the numerical solution fails (i.e., the conduit has a contact angle > $90^{\circ}$). It follows that:

$$\begin{aligned} R(n+1)=\frac{\gamma}{P(n+1)}\#\left( S29 \right) \end{aligned}$$

$$\begin{aligned} P(n+1)=P(n)+\frac{6\mu Q}{\beta(n)}\Delta x\#\left( S30 \right) \end{aligned}$$

$$\begin{aligned} P(n+1)=\frac{2\gamma h_{max}(n)}{a^{2}+h_{max}^{2}(n)}+\frac{6\mu Q}{\beta(n)}\Delta x\#\left( S31 \right) \end{aligned}$$

Across geometries where $a\gg h_{max}$, there is good agreement between semi-analytical and numerical solutions (see later in Fig. S5).

**Pendant-drop tensiometry**

To determine the interfacial tension (IFT) between the various media used in this study and FC40, we used the First Ten Angstrom 1000B Manual Drop Shape Analyzer (Model B 23A 110) plus a Point Grey Firefly MV USB camera to record drops of FC40 formed in a cuvette of medium and infer the IFT through image analysis. To do so, a 34G needle (Adhesive Dispensing Ltd) is connected to a 50 µL glass syringe (Hamilton) via PTFE tubing. The syringe is filled with FC40 and loaded onto a syringe pump (Harvard Apparatus). The needle is then lowered inside a cuvette filled with medium. A drop of FC40 is then set by infusing a desired volume through the needle. A picture of the formed drop is taken, and the software determines the IFT. Drops were imaged for 5 h, and the IFT obtained by averaging values recorded 2.5 – 5 h after starting imaging, and then averaged again over the number of repeats (3 for each medium; Fig. S3). The values obtained are $\gamma_{FBS}=22.8\pm0.44$ mN/m (DMEM + 10% FBS), $\gamma_{SR}=22.2\pm0.99$ mN/m (DMEM + 20% SR), and $\gamma_{beads}=20.7\pm0.58$ mN/m (DMEM + 10% FBS + beads). The system was also calibrated with water (a drop of water in a cuvette of FC40), yielding $\gamma_{H_{2}0}=51.8\pm0.74$ mN/m, which agrees with values accepted in the literature.


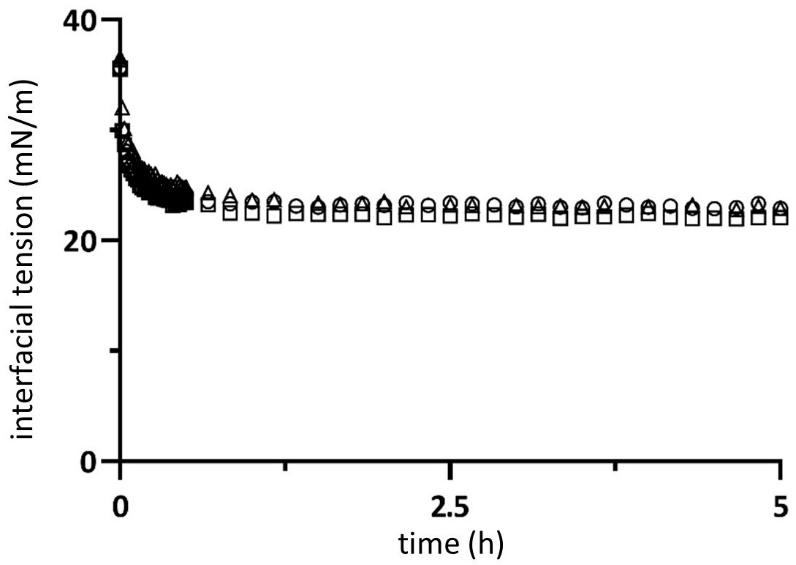


**Fig. S3. Interfacial tension measurements between FC40 and DMEM + 10% FBS.** Drops of FC40 were submerged in DMEM + 10% FBS, and the IFT measured over 5 h. Results of three repeats are shown, with each symbol corresponding to a repeat. In each repeat, IFT falls in the first 30 min to a plateau; the average value ($\gamma_{FBS}$) collected between 2.5 – 5 h was 22.8 ± 0.44 mN/m.

**Reynolds number in fluid-walled conduits**

Flow through conduits can be characterized by the Reynolds number, $Re$:

$$\begin{aligned} Re=\frac{\rho u_{avg}d_{h}}{\mu}\#\left( S32 \right) \end{aligned}$$

where $d_{h}$ is the hydraulic diameter (often used as the characteristic length for flow through non-circular ducts or pipes in lieu of the traditional circular diameter). A duct’s hydraulic diameter is:

$$d_{h}=\frac{4A}{P}$$

where $A$ is cross sectional area and $P$ is wetted perimeter. For fluidic conduits in this study, the hydraulic diameter is:

$$d_{h}=\frac{2\left( R^{2}{sin}^{-1} \left( \frac{a}{R} \right)-ab \right)}{R{sin}^{-1} \left( \frac{a}{R} \right)+a}$$

Table S1 gives Reynolds numbers calculated with Eq. S32 using experimental data from Fig. 3i.

**Table S1. Reynolds number in fluid-walled conduits**

| **flow rate**  [µL/h] | **distance from conduit exit**  [mm] | $\boldsymbol{A}$  [mm^2^] | $\boldsymbol{d}_{\boldsymbol{h}}$  [µm] | $\boldsymbol{u}_{\boldsymbol{avg}}$  [mm/s] | $\boldsymbol{Re}$ |
| --- | --- | --- | --- | --- | --- |
| 6.25 | 0 | 0.0079 | 23.990 | 0.2216 | 0.0056 |
|  | 25 | 0.0295 | 88.846 | 0.0591 | 0.0056 |
| 12.5 | 0 | 0.0100 | 30.647 | 0.3457 | 0.0112 |
|  | 25 | 0.0321 | 96.706 | 0.1080 | 0.0111 |
| 25 | 0 | 0.0109 | 33.307 | 0.6360 | 0.0225 |
|  | 25 | 0.0412 | 122.726 | 0.1685 | 0.0219 |
| 50 | 0 | 0.0127 | 38.626 | 1.0976 | 0.0450 |
|  | 25 | 0.0518 | 152.258 | 0.2682 | 0.0433 |
| 100 | 0 | 0.0153 | 46.596 | 1.8162 | 0.0898 |
|  | 25 | 0.0618 | 178.807 | 0.4496 | 0.0853 |

**Conduit capillary length**

Throughout this study, it is assumed that gravity has a negligible effect on conduit geometry as interfacial forces dominate at the microscale. The Bond number ($Bo$), a dimensionless quantity representing the ratio of gravitational to interfacial forces acting on a fluidic system, gives the relative importance of these forces:

$$\begin{aligned} Bo=\frac{\Delta\rho gL^{2}}{\gamma}\#\left( S33 \right) \end{aligned}$$

Here, $\Delta\rho$ denotes the difference in density between medium in the conduit and the overlaying FC40, and $L$ some characteristic length. A system’s capillary length $L_{c}$ corresponds to the characteristic length at which $Bo=1$ indicating that gravitational forces begin to dominate. For conduits in this study, $L_{c}=1.6$ mm. Therefore, to neglect gravity, the characteristic length must be much less than the capillary length (i.e., $L\ll L_{c}$). This can be determined by investigating the Bond number, which can be written as the ratio of hydrostatic to Laplace pressures:

$$\begin{aligned} Bo=\frac{\Delta\rho gh_{max}}{\frac{\gamma}{R}}\#\left( S34 \right) \end{aligned}$$

Using the assumption that $a\gg h_{max}$, the radius of curvature is simplified as $R=\frac{a^{2}}{2h_{max}}$ yielding:

$$\begin{aligned} Bo=\frac{\Delta\rho ga^{2}}{\gamma}\#\left( S35 \right) \end{aligned}$$

Comparing this to Eq. S33, it is easy to see the characteristic length is $L=a$. As the largest half-width in this study is $a\sim0.6$ mm (Fig. 3ii; 2.5 times smaller than $L_{c}$), this corresponds to $Bo\sim0.1$, showing that interfacial forces dominate and that gravity forces are negligible.

**Semi-analytical solution for perfect slip at the media:FC40 interface**

The semi-analytical solution is derived with the assumption of no-slip at the media:FC40 interface. This is analogous to assuming that the ratio of the viscosities of FC40 and medium is infinite such that $\lim_{\mu_{FC40}\to\infty} \frac{\mu_{FC40}}{\mu_{media}}=\infty$. To predict flow fields in conduits with any possible ratio of dynamic viscosities, we consider the other extreme in which perfect slip exists, in absence of any Marangoni effect, between immiscible fluids such that $\lim_{\mu_{FC40}\to0} \frac{\mu_{FC40}}{\mu_{media}}=0$.

The steps to derive the semi-analytical solution in such conditions mirror those outlined for the no-slip condition, and vary only by the boundary conditions applied. These are:

$$@y=\frac{h_{max}}{2}; \frac{du}{dy}=0 and @y=-\frac{h_{max}}{2}; u=0$$

Applying these conditions to the Navier-Stokes Eq. S2 yields:

$$\begin{aligned} h_{max}(x)=\left( \frac{6.5Q\mu ax}{\gamma}+h_{0}^{4} \right)^{0.25}\#\left( S36 \right) \end{aligned}$$

Slip and no-slip solutions are compared in Fig. S4.


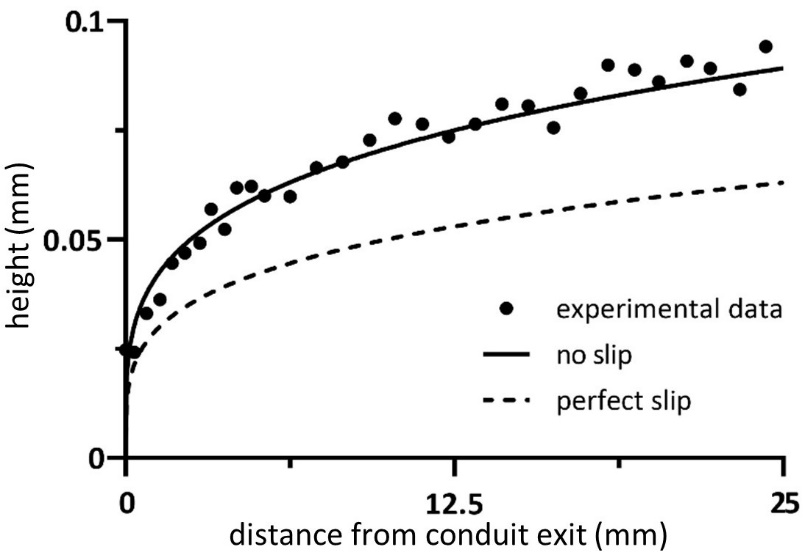


**Fig. S4. Perfect slip versus no-slip semi-analytical solutions.** Solutions are computed using Eq. S20 (no-slip) and Eq. S36 (slip; exit height assumed negligible) for a conduit (width 654 µm) perfused at 25 µL/h. Experimental data (reproduced from Fig. 3i) fit the no-slip condition.

**Conduit failure**

Conduits can withstand up to a maximum flow rate ($Q_{max}$) before reaching capillary instability which occurs when the contact angle exceeds $90^{\circ}$ (defined as $\theta=\sin^{-1} \left( \frac{2ah_{max}}{a^{2}+h_{max}^{2}} \right)$). This instability occurs when half-width equals maximum height ($a=h_{max}$), producing a perfect half-circular cross-section (Fig. S5).


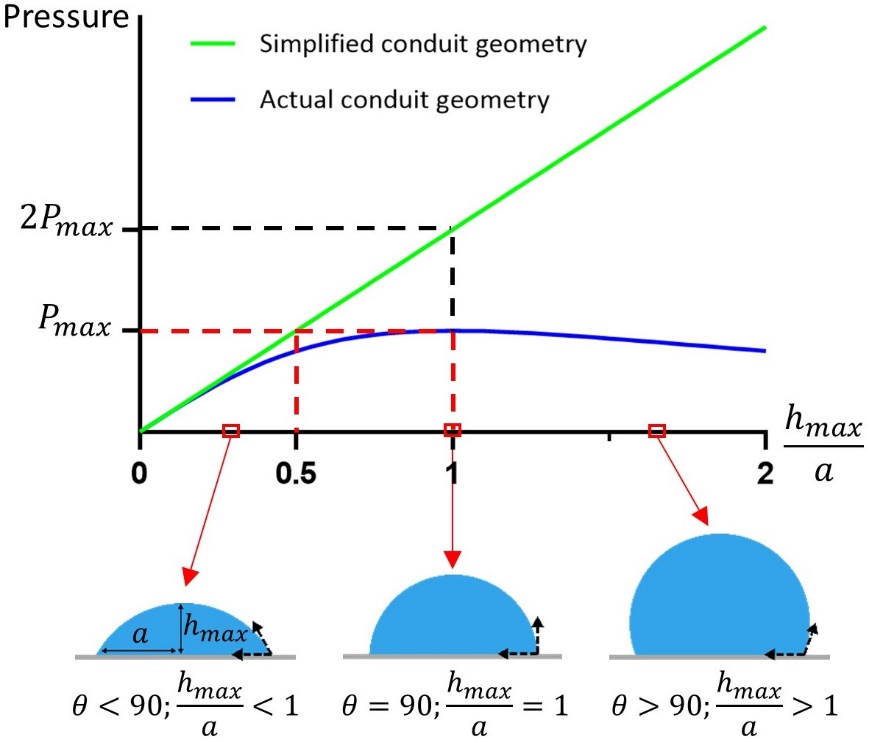


**Fig. S5. Comparing pressures calculated using the simplified (green line) and exact numerical solutions (blue curve).** For conduits with $\frac{h_{max}}{a}<0.2$, the percent error between solutions is $\leq4\%$, and – as $a\gg h_{max}$ – flow can be modelled as Poiseuille flow between two infinite parallel plates. As $\frac{h_{max}}{a}$ increases, this simplification becomes invalid and the two solutions deviate. The maximum pressure is reached when $\frac{h_{max}}{a}=1$; thereafter, pressure decreases as $\frac{h_{max}}{a}$ increases above unity.

Flow through conduits relies on a negative pressure gradient in the direction of flow. The maximum pressure a conduit can withstand occurs when the contact angle reaches $\theta=90^{\circ}$; beyond this point, flow further upstream begins to pool creating a bulging conduit where $\theta>90^{\circ}$. This then leads to pinning-line failure (and the fluid wall breaks). The maximum flow rates achievable in conduits will depend on circuit geometry (i.e., conduit length and width) and fluids used. Conduit failure will occur at the point of highest pressure in a straight conduit, and this will always be the inlet to the conduit. From the exact numerical model (blue curve in Fig. S5), we see that the maximum pressure is reached when $h_{max}=a$, and that this value can be extrapolated onto the simplified semi-analytical solution (Eq. S20, green curve in Fig. S5) when $h_{max}=0.5a$. This $h/a$ ratio is the limit at which the maximum flow rate is determined using the semi-analytical solution:

$$\begin{aligned} Q_{max}=\frac{\gamma}{26.08\mu ax}h_{max}^{4} \end{aligned}$$

$$\begin{aligned} \therefore Q_{max}=\frac{\gamma a^{3}}{417.28\mu x}\#\left( S37 \right) \end{aligned}$$

Fig. S5 highlights $\frac{h_{max}}{a}$ ratios where the simplified semi-analytical solution can be reliably used, which corresponds to $\frac{h_{max}}{a}\leq0.2$, at which point there is $\leq4\%$ error between semi-analytical and numerical solutions.

**Collapsing numerical and semi-analytical solutions**

There is good agreement between numerical and semi-analytical predictions of conduit properties for geometries investigated here. These theoretical projections can be collapsed onto a reference curve $h_{max,ref}$ by raising $Q, a$ and $\mu$ to the 0.25 power and $\gamma$ to the -0.25 power (see Equation S20) such as:

$$\begin{aligned} \frac{h_{max}}{h_{max,ref}}=\left( \frac{Q}{Q_{ref}} \right)^{0.25}\left( \frac{a}{a_{ref}} \right)^{0.25}{\left( \frac{\mu}{\mu_{ref}} \right)^{0.25}\left( \frac{\gamma}{\gamma_{ref}} \right)}^{-0.25}\#\left( S38 \right) \end{aligned}$$

This equation accurately predicts the height profile of a conduit for any given volumetric flow rate, width, dynamic viscosity, and interfacial tension.

As our model is simplified by certain assumptions, we looked at their effect on predictions by comparing the semi-analytical model with the numerical model of the conduit (Fig. S6). Both models agree well with each other for conduit lengths of up to 100 mm, after which they diverge, differing by 6.5% at 300 mm. However, our conduits are typically ≤ 25 mm as they are printed on 6 cm dishes, so divergence is tiny.


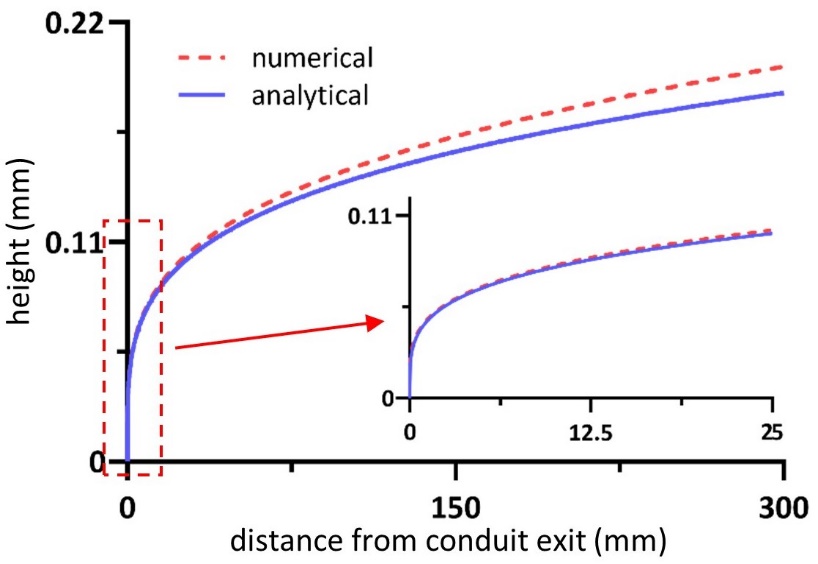


**Fig. S6. Divergence of numerical and semi-analytical solutions.** Heights were calculated using the iterative approach detailed in Eq. S21-31 (numerical solution), or Eq. S20 (semi-analytical solution), for a conduit with width 1 mm and flow rate 25 µL/h. Models diverge as conduit length increases, with a difference of 6.5% in conduit height 300 mm from the exit. For conduit lengths ≤25 mm relevant here (inset), the two models yield essentially similar results.

**Error analysis for conduit-height predictions**

To determine whether height measurements agree with theoretical predictions within an acceptable margin of error, the Root Sum of the Squares (RSS) error propagation method was used to calculate associated measurement uncertainty:

$$\varepsilon_{calc}=\sqrt{\left( \frac{\partial h_{max}}{\partial Q}\sigma_{Q} \right)^{2}+\left( \frac{\partial h_{max}}{\partial\mu}\sigma_{\mu} \right)^{2}+\left( \frac{\partial h_{max}}{\partial a}\sigma_{a} \right)^{2}+\left( \frac{\partial h_{max}}{\partial x}\sigma_{x} \right)^{2}+\left( \frac{\partial h_{max}}{\partial\gamma}\sigma_{\gamma} \right)^{2}}$$

Using u-substitution, the partial derivatives above (also known as sensitivity factors) are computed as follows:

$$\frac{\partial h_{max}}{\partial Q}=\frac{26.08\mu ax}{4\gamma\left( \frac{26.08Q\mu ax}{\gamma} \right)^{0.75}}$$

$$\frac{\partial h_{max}}{\partial\mu}=\frac{26.08Qax}{4\gamma\left( \frac{26.08Q\mu ax}{\gamma} \right)^{0.75}}$$

$$\frac{\partial h_{max}}{\partial a}=\frac{26.08Q\mu x}{4\gamma\left( \frac{26.08Q\mu ax}{\gamma} \right)^{0.75}}$$

$$\frac{\partial h_{max}}{\partial x}=\frac{26.08Q\mu a}{4\gamma\left( \frac{26.08Q\mu ax}{\gamma} \right)^{0.75}}$$

$$\frac{\partial h_{max}}{\partial\gamma}=\frac{26.08Q\mu ax}{4\gamma^{2}\left( \frac{26.08Q\mu ax}{\gamma} \right)^{0.75}}$$

Next, the uncertainty of each individual variable ($\sigma$) is determined:

- $\sigma_{Q}$ is the transducer uncertainty of the Harvard PhD Ultra syringe pump and from the user manual the pump’s flow rate accuracy is $\pm0.25\%$ ($\sigma_{q}=0.0025Q$ µL/h).
- $\sigma_{\mu}$ is the standard deviation of dynamic viscosities taken from 10 separate solutions of DMEM + 10% FBS measured with a Hydramotion Viscolite 700 portable viscometer ($\sigma_{\mu}=0.05$ mPa.s).
- $\sigma_{a}$ is the standard deviation of conduit half-width along its 25 mm length (i.e., the error associated with jetting). For measurements performed on the Zeiss Axio Observer microscope, widths were measured at 35 distinct locations ($\sigma_{a}=8$ µm).
- $\sigma_{x}$ is determined by the microscope’s specifications; for the Zeiss Axio Observer microscope, the accuracy of the stage in the x, y-plane corresponds to $\pm1$ µm ($\sigma_{x}=1$ µm).
- $\sigma_{\gamma}$ is unknown as interfacial tension is determined by fitting the semi-analytical solution to experimental data, and thus error in interfacial tension is not included.

Finally, error due to experimental uncertainty from the measurement of conduit height using beads ($\varepsilon_{bead}$) must also be included. This uncertainty takes into account the acquisition error ($\sigma_{bead}$) and twice bead diameter ($2d_{bead}$):

$$\varepsilon_{bead}=\sqrt{\sigma_{bead}^{2}+2d_{bead}^{2}}$$

To measure acquisition error, the distance between a single pair of beads (one on the surface of the dish and one on the media:FC40 interface) was measured 15 times and a standard deviation was calculated such that $\sigma_{bead}=0.9$µm. Unlike the calculated expanded sensitivity $\varepsilon_{calc}$ which depends on location (i.e., the expected error increases as distance from the exit increases), $\varepsilon_{bead}$ is a fixed quantity applied along the entire conduit such that the total propagated error is:

$$\varepsilon_{total}=\sqrt{\varepsilon_{calc}^{2}+\varepsilon_{bead}^{2}}$$

Values for $\varepsilon_{total}$ associated with height predictions are given in Fig. S7.


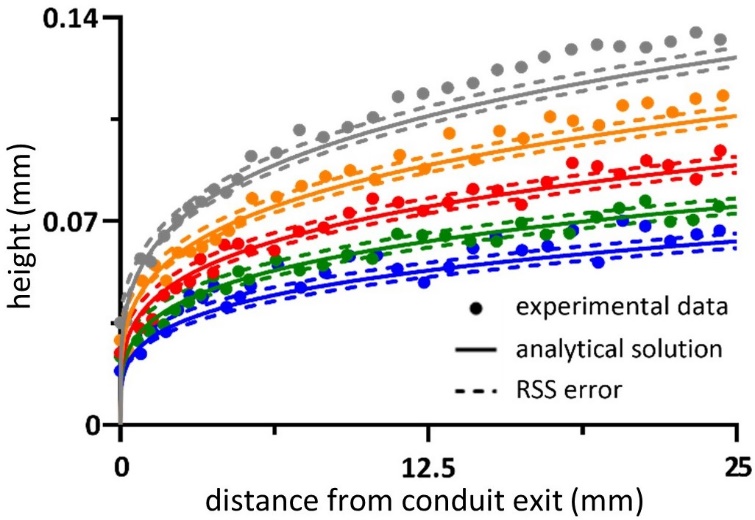


**Fig. S7. Error (root sum of squares, RSS) propagation for semi-analytical predictions of conduit heights.** Height predictions were calculated for conduits illustrated in Figure 3i (using Eq. S20) and errors ($\varepsilon_{total}$ ) determined (dashed lines bordering each coloured line – colour coding as in Fig. 3i).

**Varying flow regimes**

We studied the effect of flow regime on conduit height. First, we left a conduit at rest (no flow) for 24 h and took measurements the next day after starting flow. We then perfused a conduit overnight (for 24 h) and measured heights the following day. Comparing results with those obtained with freshly-printed conduits (Fig. 3i), although the variance of height over time is small between experiments (Fig. S8), the dynamic nature of interfacial tension could be expected to introduce a time-dependent variable to explain the small differences measured.


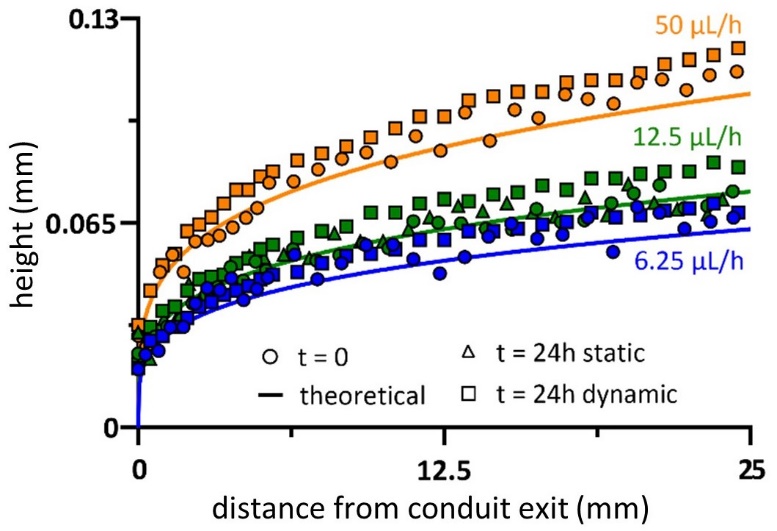


**Fig. S8. Properties of fluid walls remain stable across different flow regimes.** Heights in conduits experiencing different regimes were measured and compared with theoretical predictions (using Eq. 2). A conduit (width ~650 µm) was printed, left for 24 h without flow, then perfused at 12.5 µL/h, and heights measured (‘t = 24 h static’). Another conduit was printed and infused for 24 h at 12.5 µL/h before measuring heights (‘t = 24h dynamic’). Heights were also measured after decreasing flow to 6.25 µL/h, and then increasing it to 50 µL/h. Comparing these data with those in Fig. 3i (‘t = 0’) we find the different regimes had little effect on height.

**Splitting conduits**

In Fig. 5, we looked at the effect of one parent conduit splitting into two identical daughter conduits, and determined from continuity that flow through daughters is half the initial flow through the parent. This holds true generally for parents that split into $n$ daughters so long as the geometry of daughters is identical. Then, $Q_{d}=\frac{Q_{p}}{n}$.

However, it is more difficult to predict flows through daughters of differing geometries. Consider Fig. S9 where some branches have different half widths $a$ or length $x$.


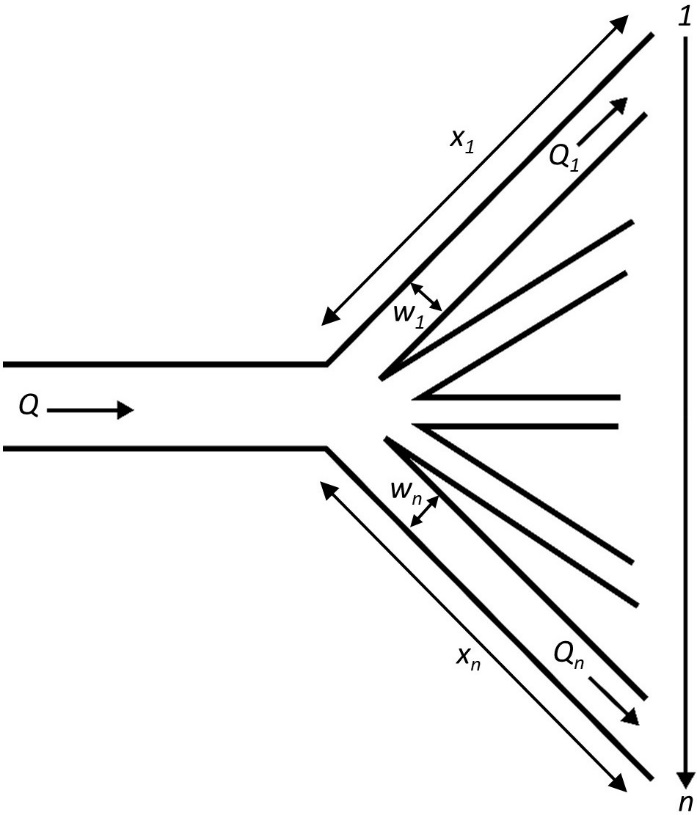


**Fig. S9. Single parent conduit splitting into multiple daughter conduits.** Each daughter conduit has a different length $x$ or width $w$ (half-width $a=\frac{w}{2}$), and therefore the resultant flow rate $Q$ through each conduit will be different and determined by Eq. S39.

As the pressure $P_{in}$ at the branch point must be the same for each branch, and since conduits are open ended (such that $P_{0}=0$), then $P_{in}-P_{0}=\Delta P$ must be the same across each branch. From our scale analysis of the effect of exit height ($h_{0}$) on conduit shape, we know $h_{0}$ can be ignored if conduits are > 1 mm in length such that:

$$\begin{aligned} P_{x}=\left( \frac{417.28\gamma^{3}\mu Qx}{a^{7}} \right)^{0.25} \end{aligned}$$

Assuming each branching conduit is > 1 mm, the pressure drop in each must be the same, and we can equate the pressure gradient in any branch $n$ to reference branch $1$ as $P_{n}=P_{1}$:

$$\left( \frac{417.4\gamma^{3}\mu Q_{n}x_{n}}{{a_{n}}^{7}} \right)^{0.25}=\left( \frac{417.4\gamma^{3}\mu Q_{1}x_{1}}{{a_{1}}^{7}} \right)^{0.25}$$

$$\frac{Q_{n}x_{n}}{{a_{n}}^{7}}=\frac{Q_{1}x_{1}}{{a_{1}}^{7}}$$

$$Q_{n}=\frac{Q_{1}{a_{n}}^{7}x_{1}}{{a_{1}}^{7}x_{n}}$$

Applying mass conservation, we know the input flow rate $Q$ must equal the sum of all flow through branches (from 1 to $n$):

$$Q=\sum_{1}^{n} Q_{n}=\sum_{1}^{n} \frac{Q_{1}{a_{n}}^{7}x_{1}}{{a_{1}}^{7}x_{n}}$$

We can remove $Q_{1}, x_{1},$ and $a_{1}$ from the summation term as these are used as reference values for all other channels and are fixed:

$$Q=\frac{nQ_{1}x_{1}}{n{a_{1}}^{7}}\sum_{1}^{n} \frac{{a_{n}}^{7}}{x_{n}}=\frac{Q_{1}x_{1}}{{a_{1}}^{7}}\sum_{1}^{n} \frac{{a_{n}}^{7}}{x_{n}}$$

This gives:

$$\begin{aligned} Q_{1}=\frac{Q{a_{1}}^{7}}{x_{1}\sum_{1}^{n} \frac{{a_{n}}^{7}}{x_{n}}}\#\left( S39 \right) \end{aligned}$$

Since the reference conduit can be any of the daughter conduits, we can apply this equation to calculate flow fields through any conduit knowing only input flow rate $Q$ and the geometrical properties of each conduit.

**Height correction for apparent depth in microscope measurements**

To determine conduit height using an inverted microscope, we first focus on a fluorescent bead at the media-FC40 interface. The height is then taken as the distance $\Delta s$ by which the stage moves to focus on a bead on the surface of the dish. However, due to mismatches between the refractive indices of the medium containing the bead (water), and the medium surrounding the microscope objective (air), the focal position of the bead does not follow the axial movement of the stage. Rather, $\Delta s$ appears smaller than reality.

To correct for this focal shift, consider Fig. S10 where the microscope stage is moved a distance $\Delta s$, and the resulting focal position is where the two red dashed lines converge, slightly below the original position of the bead. This derivation is based on a similar one presented in [1] but adds the extra layer of the polystyrene dish in the light path.


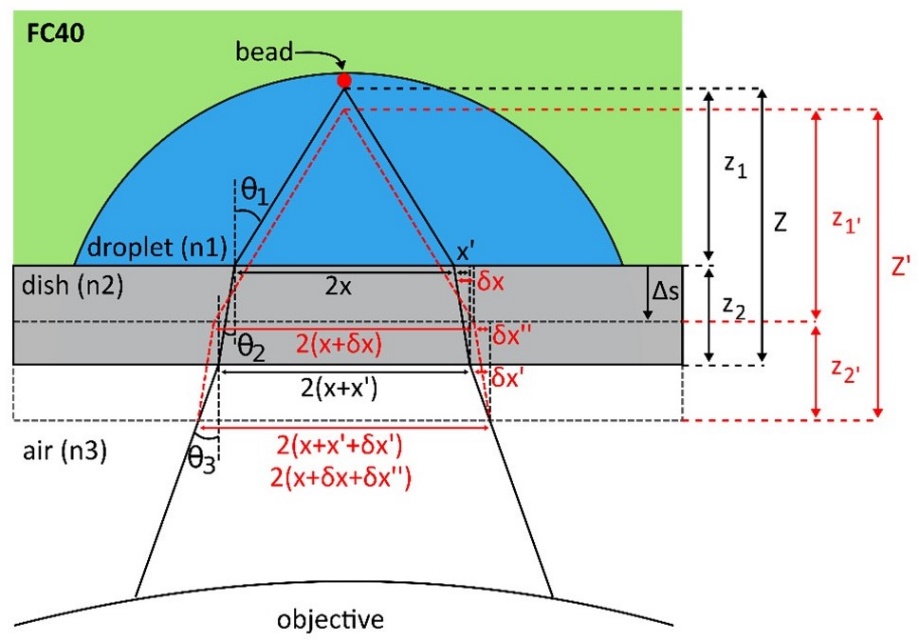


**Fig. S10. Correcting focal shift when measuring conduit height on inverted microscopes.** A drop of medium (which contains fluorescent beads and is overlaid with FC40) sits on a polystyrene dish on the stage of an inverted microscope. One red fluorescent bead stuck to the medium:FC40 interface at the peak of the spherical cap is brought into focus. Moving the stage a distance $\Delta s$ closer to the objective moves the focus below the bead by a distance $\Delta f\neq\Delta s$ due to refraction of the light path across the different substrates. Using a combination of Snell’s law and geometry, the correct value of $\Delta f$ is determined using the numerical aperture of the objective lens, the refractive index of the culture medium around the bead, and the refractive index of the air at the objective, as in Eq. S40.

The marginal rays of the light cone emerging from the bead hit the dish at angle $\theta1$, and have a half-width of $x$. Rays then travel through the dish at angle $\theta2$ and emerge from the dish into air at angle $\theta3$ with a new half-width of $x+x'$, before entering the objective. As the microscope stage is moved, we can see that the angles of the rays stay the same, while half-widths of light cones vary.

If the original depth of focus is $Z$, and moving the microscope stage results in a new depth of focus $Z'$, then the axial focal shift is given by $Z^{'}-Z$.

In Fig. S10, we have the relationships:

$$Z=z_{1}+z_{2}$$

$$Z^{'}=z_{1}^{'}+z_{2}'$$

Using trigonometry, we determine the following relations:

$$\tan\left( \theta_{1} \right)=\frac{x}{z_{1}}\equiv z_{1}=\frac{x}{\tan\left( \theta_{1} \right)}$$

$$\tan\left( \theta_{2} \right)=\frac{x'}{z_{2}}\equiv z_{2}=\frac{x'}{\tan\left( \theta_{2} \right)}$$

$$\tan\left( \theta_{1} \right)=\frac{x+\delta x}{z_{1}'}\equiv z_{1}'=\frac{x+\delta x}{\tan\left( \theta_{1} \right)}$$

$$\tan\left( \theta_{2} \right)=\frac{\delta x''}{z_{2}'}\equiv z_{2}'=\frac{\delta x''}{\tan\left( \theta_{2} \right)}$$

$$\tan\left( \theta_{3} \right)=\frac{\delta x'}{\Delta s}\equiv\delta x^{'}=\Delta s\times tan(\theta_{3})$$

As the thickness $z2$ of the dish does not change, then:

$$z_{2}=z_{2}^{'}\to\frac{x^{'}}{\tan\left( \theta_{2} \right)}=\frac{\delta x''}{\tan\left( \theta_{2} \right)}\to x^{'}=\delta x''$$

Furthermore, we can see from the half-width of the light cone emerging from the dish after the stage has moved that:

$$x+x^{'}+\delta x^{'}=x+\delta x+\delta x''$$

$$\to x+x^{'}+\delta x^{'}=x+\delta x+x'$$

$$\therefore\delta x^{'}=\delta x=\Delta s\times tan(\theta_{3})$$

The focal shift is therefore:

$$\Delta f=Z^{'}-Z=z_{1}^{'}+z_{2}^{'}-z_{1}-z_{2}$$

$$\Delta f=z_{1}^{'}-z_{1}=\frac{x+\delta x}{\tan\left( \theta_{1} \right)}-\frac{x}{\tan\left( \theta_{1} \right)}$$

$$\Delta f=\frac{\delta x}{\tan\left( \theta_{1} \right)}$$

$$\therefore\Delta f=\Delta s\frac{tan(\theta_{3})}{\tan\left( \theta_{1} \right)}$$

This can now be expressed in terms of the numerical aperture of the objective, $NA$:

$$NA=n1\times\sin\left( \theta_{1} \right)\leftrightarrow\theta_{1}={sin}^{-1} \left( \frac{NA}{n1} \right)$$

Therefore:

$$\begin{aligned} \Delta f=\Delta s\frac{tan({sin}^{-1} \left( \frac{NA}{n3} \right)}{tan({sin}^{-1} \left( \frac{NA}{n1} \right)}\#\left( S40 \right) \end{aligned}$$

For small $NA$, we use the small-angle approximation:

$$\sin\left( \theta\right)=\theta,\tan\left( \theta\right)=\theta$$

and:

$$\Delta f=\Delta s\frac{tan\left( \frac{NA}{n3} \right)}{tan\left( \frac{NA}{n1} \right)}=\Delta s\frac{n1}{n3}$$

To confirm the accuracy of Eq. S40 for height corrections, we measured the heights of sessile drops of known volumes with the Olympus microscope. Droplets of 0.5 and 1 µL were deposited on polystyrene culture dishes, made from the same media + bead mixture used to measure conduit heights. As before, the diameter and heights of drops were measured, theoretical heights determined using the known volume and measured footprint. The heights measured using beads were then corrected using Eq. S40 and show good agreement with theory (using the height-to-volume equation for sessile drops [2]; average of 2% difference between theoretical and corrected heights across 21 individual measurements; Table S2).

**Table S2. Comparison between sessile-drop height measurements and theoretical predictions using Eq. S40**

| **drop volume (µL)** | **footprint diameter (mm)** | **measured height (mm)** | **theoretical height (mm)** | **corrected**  **height (mm)** | **ratio corrected vs theoretical** |
| --- | --- | --- | --- | --- | --- |
| 0.5 | 1.666 | 0.294 | 0.422 | 0.427 | 1.012 |
|  | 1.670 | 0.278 | 0.421 | 0.404 | 0.960 |
|  | 1.663 | 0.270 | 0.424 | 0.392 | 0.926 |
|  | 1.667 | 0.287 | 0.422 | 0.417 | 0.989 |
|  | 1.678 | 0.295 | 0.418 | 0.429 | 1.026 |
|  | 1.685 | 0.290 | 0.415 | 0.421 | 1.016 |
|  | 1.680 | 0.262 | 0.417 | 0.381 | 0.913 |
|  | 1.687 | 0.252 | 0.414 | 0.366 | 0.884 |
|  | 1.696 | 0.273 | 0.411 | 0.397 | 0.966 |
| 1 | 2.058 | 0.368 | 0.549 | 0.535 | 0.974 |
|  | 2.078 | 0.377 | 0.541 | 0.548 | 1.013 |
|  | 2.101 | 0.370 | 0.532 | 0.538 | 1.012 |
|  | 2.136 | 0.376 | 0.518 | 0.546 | 1.056 |
|  | 2.134 | 0.345 | 0.518 | 0.501 | 0.967 |
|  | 2.136 | 0.334 | 0.518 | 0.485 | 0.938 |
|  | 2.165 | 0.315 | 0.506 | 0.458 | 0.904 |
|  | 2.157 | 0.350 | 0.509 | 0.509 | 0.999 |
|  | 2.167 | 0.338 | 0.506 | 0.491 | 0.972 |
|  | 2.113 | 0.363 | 0.527 | 0.528 | 1.001 |
|  | 2.126 | 0.350 | 0.521 | 0.509 | 0.975 |
|  | 2.138 | 0.370 | 0.517 | 0.538 | 1.040 |

**Maintaining constant shear along fluid-walled conduits**

For conduits with chokes, the profile of both choke and conduit are determined using Eq. 2. While the exit height of the choke can be assumed to be zero, the conduit-choke junction determines conduit exit height ($x_{conduit}=0$) as this will naturally be larger than the choke’s starting height ($x_{choke}=L_{choke}$). We assume the transition between the choke start to conduit exit is small so the Laplace pressure is constant, and that the pressure-head difference due to FC40 is negligible; then, we equate the Laplace pressure in both regions such that $\Delta P_{conduit(x = 0)}=\Delta P_{choke(x = L)}$ and hence $R_{conduit(x = 0)}=R_{choke(x = L)}$. As the radius of curvature $R=\frac{a^{2}+h^{2}}{2h}$, we solve for conduit starting-height yielding:

$$h_{conduit\left( x = 0 \right)}=\frac{a_{choke}^{2}+h_{choke\left( x=L \right)}^{2}-\sqrt{\left( a_{choke}^{2}+h_{choke\left( x=L \right)}^{2} \right)^{2}-{4a}_{conduit}^{2}h_{choke\left( x = L \right)}^{2}}}{2h_{choke\left( x=L \right)}}$$

Here, $a$ is fixed and defined when printing the conduit, and $h_{choke\left( x=L \right)}$ is the height of the choke before the junction and is calculated from the semi-analytical solution. Since the junction has a saddle shape, for conduits in Fig. 4B,C, the height of the choke, $h_{choke(x=L)}$, is taken at a distance away from the junction equivalent to one choke width (e.g., for choke width = 400 µm, $L_{choke}=10$ mm, $h_{choke\left( x=L \right)}=9.6$ mm). For conduits with geometries where $a_{conduit}\gg h_{conduit}$ and $a_{choke}\gg h_{choke}$, the radius of curvature is simplified to $R=\frac{a^{2}}{2h}$ and thus:

$$h_{conduit\left( x = 0 \right)}=\frac{a_{conduit}^{2}h_{choke(x = L)}}{a_{choke}^{2}}$$

In this case, introducing a choke has the effect of increasing $h_{conduit}$ proportionately to $\frac{a_{conduit}^{2}}{a_{choke}^{2}}$, and maintains the conduit at an approximately constant height upstream of the choke due to the small pressure drop.

**Semi-analytical solution for shear stress**

We evaluate the wall shear stress at any location along conduit length and any z-location across its width. Starting from Eq. S6 and substituting $u_{max}$ with Eq. S10 yields:

$$\begin{aligned} u_{max(z)}=\frac{Q{h_{z}}^{2}}{{{0.6h}_{max}}^{3}a}\#\left( S41 \right) \end{aligned}$$

Since $\tau=\mu\frac{du}{dy}$, and differentiating $u\left( y \right)=\left( 1-\frac{4y^{2}}{h_{max}^{2}} \right)u_{max}$ to obtain $\frac{du}{dy}=\frac{8y}{h_{max}^{2}}u_{max}$, we express the shear stress in the conduit as:

$$\begin{aligned} \tau_{max(z)}=\frac{8\mu y}{{h_{z}}^{2}}u_{max(z)}\#\left( S42 \right) \end{aligned}$$

Substituting $u_{max(z)}$ from Eq. S41 into Eq. S42 we get:

$$\begin{aligned} \tau_{max(z)}=\frac{8Q\mu}{{{0.6h}_{max}}^{3}a}y \end{aligned}$$

Wall shear stress is evaluated at $y=\frac{h_{z}}{2}$ hence:

$$\begin{aligned} \tau_{max(z)}=\frac{4Q\mu h_{z}}{{{0.6h}_{max}}^{3}a}\#\left( S43 \right) \end{aligned}$$

At the conduit centre ($z=0$), $h_{z}=h_{max}$, therefore Eq. S43 becomes:

$$\begin{aligned} \tau_{\max\left( 0 \right)}=\frac{4Q\mu}{0.6h_{max}^{2}a}\#\left( S44 \right) \end{aligned}$$

Expressing $\tau_{\max\left( z \right)}$ in terms of $\tau_{\max\left( 0 \right)}$ using Eq. S44:

$$\begin{aligned} \tau_{max(z)}=\tau_{max(0)}\frac{h_{z}}{h_{max}}\#(S45) \end{aligned}$$

Now evaluating $\tau_{max}$ by substituting $h_{max}$ with Eq. S20 finally yields:

$$\begin{aligned} \tau_{max(z)}=1.28\sqrt{\frac{\mu\gamma Q}{a^{3}x}}\frac{h_{z}}{h_{max}}\#(S46) \end{aligned}$$

**Effect of a choke on the gradient of wall shear stress**

The addition of a choke creates a region of nearly uniform shear upstream of the choke (i.e., $\frac{d\tau}{dx}$ is very small). This shear distribution would be achieved in a conduit without choke, but at a much farther distance. The choke allows users to ‘skip ahead’ through a conduit to reach a desired shear stress distribution. Conduits with small $\frac{d\tau}{dx}$ can be obtained at operational scales (that fit in 60 mm dishes) that would require much longer lengths of conduit otherwise. To investigate the order-of-magnitude effect of a choke on $\frac{d\tau}{dx}$, consider the two systems in Fig. S11.


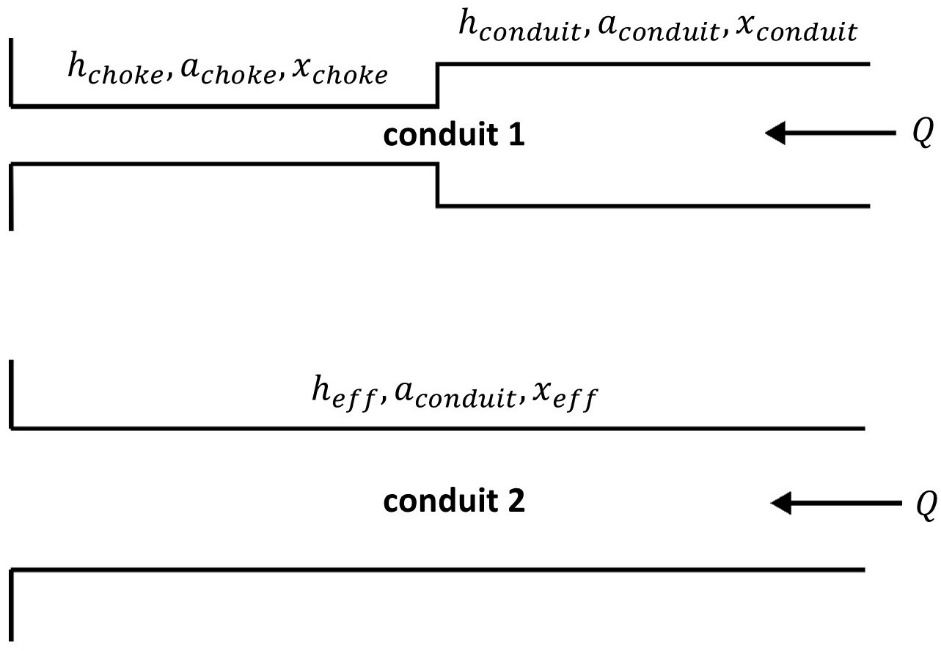


**Fig. S11. Conduits with and without a choke.** Both conduits are perfused at the same flow rate $Q$, and $a_{conduit}$ in conduit 1 is equivalent to $a_{conduit}$ in conduit 2.

Due to the choke in conduit 1, $h_{conduit}\approx h_{choke}\left( \frac{a_{conduit}}{a_{choke}} \right)^{2}$. From our semi-analytical equation:

$$h_{choke}=\left( \frac{26.08Q\mu a_{choke}x_{choke}}{\gamma} \right)^{0.25}$$

$$\begin{aligned} \therefore h_{conduit}\approx\left( \frac{26.08Q\mu a_{choke}x_{choke}}{\gamma} \right)^{0.25}\left( \frac{a_{conduit}}{a_{choke}} \right)^{2}\#\left( S47 \right) \end{aligned}$$

Next, for the same flow rate we determine the distance $x_{eff}$ that conduit 2 (which has width $a_{conduit}$) would need to reach so that $h_{eff}=h_{conduit}$ at the junction between conduits (i.e., the effective length introduced by the choke):

$$\left( \frac{26.08Q\mu a_{conduit}x_{eff}}{\gamma} \right)^{0.25}=\left( \frac{26.08Q\mu a_{choke}x_{choke}}{\gamma} \right)^{0.25}\left( \frac{a_{conduit}}{a_{choke}} \right)^{2}$$

$$a_{conduit}x_{eff}=a_{choke}x_{choke}\left( \frac{a_{conduit}}{a_{choke}} \right)^{8}$$

$$\begin{aligned} \therefore x_{eff}=x_{choke}\left( \frac{a_{conduit}}{a_{choke}} \right)^{7}\#\left( S48 \right) \end{aligned}$$

As an example, if $x_{choke}=1$ mm, $a_{choke}=0.2$ mm, and $a_{conduit}=0.5$ mm, then $x_{eff}=0.6$ m.

Next, we evaluate the relationship between $\frac{d\tau}{dx}$ and the distance $x$ along the conduit. Shear stress in the conduit ($@h_{max(x)}$) is:

$$\begin{aligned} \tau=1.28\left( \frac{Q\mu\gamma}{a^{3}x} \right)^{0.5}\#\left( S49 \right) \end{aligned}$$

$$\begin{aligned} \therefore\tau\propto\left( \frac{1}{a^{3}x} \right)^{0.5} \end{aligned}$$

As:

$$\frac{d}{dx}\left( \left( \frac{1}{a^{3}x} \right)^{0.5} \right)=-\frac{1}{2}a^{3}\left( \frac{1}{a^{3}x} \right)^{\frac{3}{2}}$$

Then:

$$\begin{aligned} \frac{d\tau}{dx}\propto{a^{3}\left( \frac{1}{a^{3}x} \right)}^{\frac{3}{2}}\propto\left( \frac{1}{ax} \right)^{\frac{3}{2}}\#\left( S50 \right) \end{aligned}$$

Using Eq. S48, the shear gradient after the choke is:

$$\begin{aligned} \left( \frac{d\tau}{dx} \right)_{choke}\propto\left( \frac{1}{a_{conduit}x_{eff}} \right)^{\frac{3}{2}}\propto\left( \frac{1}{a_{conduit}x_{choke}\left( \frac{a_{conduit}}{a_{choke}} \right)^{7}} \right)^{\frac{3}{2}}\#\left( S51 \right) \end{aligned}$$

The shear gradient for a conduit without choke at $x_{choke}$ is:

$$\begin{aligned} \left( \frac{d\tau}{dx} \right)_{no choke}\propto\left( \frac{1}{a_{conduit}x_{choke}} \right)^{\frac{3}{2}}\#\left( S52 \right) \end{aligned}$$

Hence, the change in $\frac{d\tau}{dx}$ from the addition of a choke can be evaluated as:

$$\begin{aligned} \frac{\left( \frac{d\tau}{dx} \right)_{no choke}}{\left( \frac{d\tau}{dx} \right)_{choke}}\equiv\frac{\left( \frac{1}{a_{conduit}x_{choke}} \right)^{\frac{3}{2}}}{\left( \frac{1}{a_{conduit}x_{choke}\left( \frac{a_{conduit}}{a_{choke}} \right)^{7}} \right)^{\frac{3}{2}}}\equiv\left( \frac{a_{conduit}}{a_{choke}} \right)^{\frac{21}{2}}\#\left( S53 \right) \end{aligned}$$

In our example, $\frac{d\tau}{dx}$ in the conduit after the choke will be $\sim15000$ fold smaller than $\frac{d\tau}{dx}$ in the straight conduit at the same distance from the exit (equivalent to a 99.99% decrease). Table S3 gives the percent change in $\tau$ over 1 cm upstream of a choke, for various choke lengths and $\frac{a_{conduit}}{a_{choke}}$ ratios.

**Table S3. % change in** $\boldsymbol{\tau}$ **over 1 cm for varying half-width ratios and choke lengths**

| $\frac{\boldsymbol{a}_{\boldsymbol{conduit}}}{\boldsymbol{a}_{\boldsymbol{choke}}}$ | **% change in** $\boldsymbol{\tau}$ **over 1 cm with choke length of:** | | |
| --- | --- | --- | --- |
|  | **1 mm** | **5 mm** | **10 mm** |
| 2.5 | 0.81 | 0.16 | 0.08 |
| 2 | 3.69 | 0.77 | 0.39 |
| 1.25 | 43.18 | 16.07 | 9.08 |
| 1 | 69.85 | 42.26 | 29.29 |

**Error from** $\frac{\boldsymbol{h}}{\boldsymbol{a}}$ **simplification on shear stress gradient in choked conduits**

The height of a conduit after a choke is estimated without simplifications as:

$$\begin{aligned} h_{conduit(exact)}=\frac{a_{choke}^{2}+h_{choke}^{2}-\sqrt{\left( a_{choke}^{2}+h_{choke}^{2} \right)^{2}-{4a}_{conduit}^{2}h_{choke}^{2}}}{2h_{choke}}\#\left( S54 \right) \end{aligned}$$

However, Eq. S54 is simplified when assuming the radius of curvature is expressed as $R=\frac{a^{2}}{2h}$, yielding:

$$\begin{aligned} h_{conduit\left( simp \right)}=h_{choke}\left( \frac{a_{conduit}}{a_{choke}} \right)^{2}\#\left( S55 \right) \end{aligned}$$

The error or difference from this simplification is given as:

$$\begin{aligned} \frac{h_{conduit(exact)}}{h_{conduit\left( simp \right)}}=c\#\left( S56 \right) \end{aligned}$$

where $c$ is a constant dependent on $h_{choke}$, $a_{choke}$, and $a_{conduit}$. From Eq. S55, the effective length introduced by the choke is determined (as in Eq. S48):

$$\begin{aligned} x_{eff\left( simp \right)}=x_{choke}\left( \frac{a_{conduit}}{a_{choke}} \right)^{7}\#\left( S57 \right) \end{aligned}$$

Introducing Eq. S57 into the expression for wall shear stress (Eq. S49) yields:

$$\begin{aligned} \tau_{simp}=1.28\left( \frac{Q\mu\gamma}{a^{3}x_{eff}} \right)^{0.5}\#\left( S58 \right) \end{aligned}$$

The radius of curvature simplification from Eq. S55 introduces an error ($c$) in Eq. S58; this is accounted for using Eq. S56, such that $x_{eff}$ and $\tau$ become:

$$\begin{aligned} x_{eff(corrected)}=x_{choke}c^{4}\left( \frac{a_{conduit}}{a_{choke}} \right)^{7}\#\left( S59 \right) \end{aligned}$$

$$\begin{aligned} \tau_{corrected}=1.28\left( \frac{Q\mu\gamma}{a^{3}x_{eff(corrected)}} \right)^{0.5}\#\left( S60 \right) \end{aligned}$$

Finally, as in Eq. S44, $\tau$ may also be expressed in terms of conduit height rather than effective length, using the exact (Eq. S54) or simplified equation (Eq. S55). The exact expression using Eq. S54 is:

$$\begin{aligned} \tau_{exact}=\frac{4Q\mu}{0.6h_{conduit(exact)}^{2}a_{conduit}}\#\left( S61 \right) \end{aligned}$$

As shear stress in Fig. 4D is calculated using Eq. S61, we evaluate the effect of the radius of curvature simplification on $\tau$ (Eq. S58), and subsequent correction (Eq. S60), for the choked conduits in Fig. 4B. Table S4 summarizes the estimated % change in $\tau$ for these conduits over 1 cm upstream of the choke, showing equivalent results for Eq. S60 and S61.

**Table S4. Effect of** $\boldsymbol{h/a}$ **simplification on** $\boldsymbol{\tau}$ **predictions**

| **choke length** | **1 mm** | **5 mm** | **10 mm** |
| --- | --- | --- | --- |
| $\boldsymbol{h}_{\boldsymbol{conduit(exact)}}$ **(Eq. S54) [µm]** | 157 | 287 | 322 |
| $\boldsymbol{h}_{\boldsymbol{conduit(simp)}}$ **(Eq. S55) [µm]** | 152 | 220 | 237 |
| $\boldsymbol{c}$ **(Eq. S56)** | 1.03 | 1.31 | 1.36 |
| $\boldsymbol{x}_{\boldsymbol{eff(simp)}}$ **(Eq. S57) [m]** | 0.58 | 2.54 | 3.28 |
| $\boldsymbol{x}_{\boldsymbol{eff(corrected)}}$ **(Eq. 59) [m]** | 0.66 | 7.39 | 11.19 |
| $\boldsymbol{\tau}_{\boldsymbol{simp}}$ **@ choke (Eq. S58) [Pa]** | 9.76E-04 | 4.65E-04 | 3.87E-04 |
| $\boldsymbol{\tau}_{\boldsymbol{simp}}$ **@ 1 cm (Eq. S58) [Pa]** | 9.68E-04 | 4.64E-04 | 3.87E-04 |
| $\boldsymbol{d\tau/dx}$ **1cm (%)** | 0.85 | 0.20 | 0.15 |
| $\boldsymbol{\tau}_{\boldsymbol{corrected}}$ **@ choke (Eq. S60) [Pa]** | 9.13E-04 | 2.73E-04 | 2.10E-04 |
| $\boldsymbol{\tau}_{\boldsymbol{corrected}}$ **@ 1 cm (Eq. S60) [Pa]** | 9.06E-04 | 2.73E-04 | 2.10E-04 |
| $\boldsymbol{d\tau/dx}$ **1cm (%)** | 0.75 | 0.07 | 0.04 |
| $\boldsymbol{\tau}_{\boldsymbol{exact}}$ **@ choke (Eq. S61) [Pa]** | 9.31E-04 | 2.78E-04 | 2.14E-04 |
| $\boldsymbol{\tau}_{\boldsymbol{exact}}$ **@ 1 cm (Eq. S61) [Pa]** | 9.24E-04 | 2.78E-04 | 2.14E-04 |
| $\boldsymbol{d\tau/dx}$ **1cm (%)** | 0.75 | 0.07 | 0.04 |

**Culturing cells in fluid-walled conduits**

We plated human umbilical vein endothelial cells (HUVECs) in conduits (± choke) and perfused them overnight (at 25 or 50 µL/h) to look at the effect of flow on cell viability. Fig. S11 shows that after 24 h, flow had little effect on HUVEC morphology or alignment, which is to be expected from the magnitude of the shear stress in such conduits (see Fig. 4D) [3].


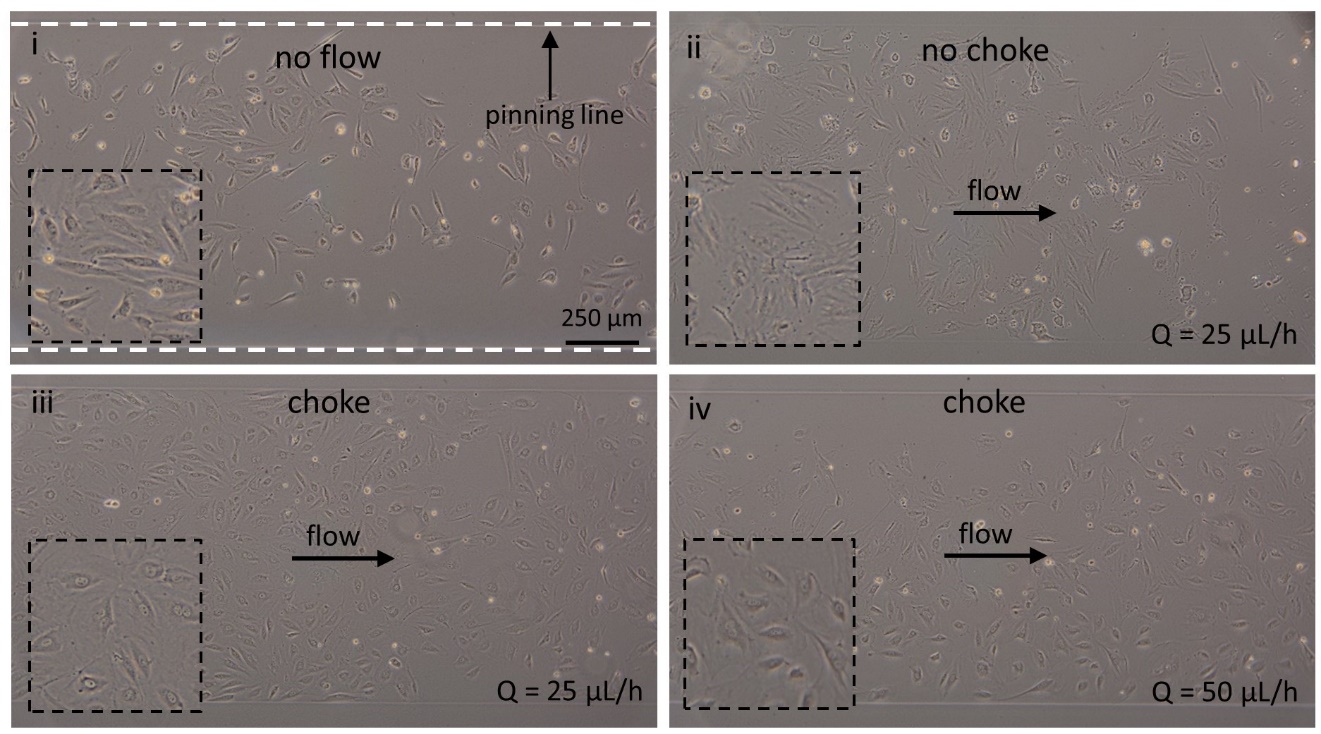


**Fig. S12. HUVECs in fluid-walled conduits.** Cells were plated in 4 conduits ± chokes (conduit width 1 mm; choke width 400 µm) and incubated overnight before flow was applied. Insets show close-ups of cells in conduits. **(i)** Control conduit without flow. White dashed lines indicate conduit pinning lines (triple contact line polystyrene-media-FC40). **(ii)** Conduit without choke, perfused at 25 µL/h. **(iii)** Conduit with choke, perfused at 25 µL/h. **(iv)** Conduit with choke, perfused at 50 µL/h.

**Movie S1. Addition of FBS to DMEM immediately induces the no-slip condition**

Two square chambers (1.9 x 1.9 mm) each containing 400 nL DMEM plus 4 mg/ml red dye (Allura Red in H_2_O, Sigma) and 3-10 µm glass beads (1:1000 dilution, Polysciences, Inc., 07666) are each surrounded by FC40 walls; these chambers sit in a dish filled with FC40 on the Olympus microscope (4x objective). Next, the jetting needle (diameter 500 µm) – held vertically by attachment to the microscope condenser, filled with FC40, and connected to a syringe pump – is lowered through the fluorocarbon until ~1 mm above the footprint of the FC40 wall between the two chambers. This needle gives the circular shadow in the middle of video images. The movie begins as the pump is started and projects a submerged FC40 jet downwards at 8 µL/s; this induces shear at the FC40:medium interface so beads in both chambers swirl rapidly. After ~10 s, 0.5 µL DMEM + 10% FBS is manually pipetted into the bottom chamber; beads in the inoculated chamber rapidly stop moving, as those in the upper one continue. This shows that FBS immediately induces a no-slip interface.

**SI References**

[1] T. D. Visser, J. L. Oud, and G. J. Brakenhoff, “Refractive index and axial distance measurements in 3-D microscopy,” *Optik (Stuttg).*, vol. 90, no. 1, pp. 17–19, 1992.

[2] E. J. Walsh *et al.*, “Microfluidics with fluid walls,” *Nat. Commun.*, vol. 8, no. 1, p. 816, 2017, doi: 10.1038/s41467-017-00846-4.

[3] J. Shemesh, I. Jalilian, A. Shi, G. Heng Yeoh, M. L. Knothe Tate, and M. Ebrahimi Warkiani, “Flow-induced stress on adherent cells in microfluidic devices,” *Lab Chip*, vol. 15, no. 21, pp. 4114–4127, 2015, doi: 10.1039/C5LC00633C.
